# 2D Cu(I)-I Coordination Polymer with Smart Optoelectronic Properties and Photocatalytic Activity as a Versatile Multifunctional Material

**DOI:** 10.1021/acs.inorgchem.3c00616

**Published:** 2023-06-30

**Authors:** María Murillo, Reinhold Wannemacher, Juan Cabanillas-González, Ulises R. Rodríguez-Mendoza, Javier Gonzalez-Platas, Akun Liang, Robin Turnbull, Daniel Errandonea, Ginés Lifante-Pedrola, Andrea García-Hernán, Jose I. Martínez, Pilar Amo-Ochoa

**Affiliations:** † Dpto. de Química Inorgánica, Universidad Autónoma de Madrid, Madrid 28049, Spain; ‡ IMDEA Nanociencia Ciudad Universitaria de Cantoblanco, Madrid 28049, Spain; § Dpto. de Física, Instituto Universitario de Nanomateriales y Nanotecnología (IMN), MALTA Consolider Team, Universidad de La Laguna, Avda. Astrofísico Fco. Sánchez s/n, La Laguna Tenerife E-38204, Spain; ∥ Dpto. de Física, Instituto Universitario de Estudios Avanzados en Física Atómica, Molecular y Fotónica (IUDEA), MALTA Consolider Team, Universidad de La Laguna, Avda. Astrofísico Fco. Sánchez s/n, La Laguna Tenerife E-38204, Spain; ⊥ Dpto de Física Aplicada-ICMUV-MALTA Consolider Team, Universitat de Valencia, c/Dr. Moliner 50, Burjassot (Valencia) 46100, Spain; # Dpto. Física Aplicada, Universidad Autónoma de Madrid, Madrid 28049, Spain; ∇ Dpto. Surfaces, Coatings and Molecular Astrophysics, Institute of Material Science of Madrid (ICMM-CSIC), University Campus of Cantoblanco, Madrid ES-28049, Spain; ○ Institute for Advanced Research in Chemical Sciences (IAdChem), Universidad Autónoma de Madrid, Madrid 28049, Spain

## Abstract

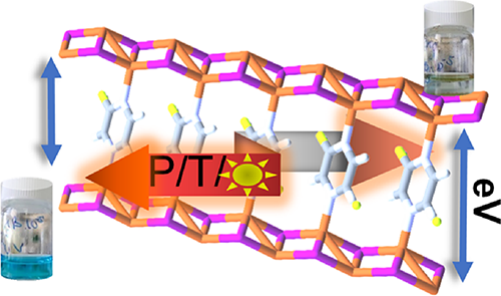

This work presents two isostructural Cu(I)-I 2-fluoropyrazine (Fpyz) luminescent and semiconducting 2D coordination polymers (CPs). Hydrothermal synthesis allows the growth of *P*-1 space group single crystals, whereas solvent-free synthesis produces polycrystals. Via recrystallization in acetonitrile, *P*2_1_ space group single crystals are obtained. Both show a reversible luminescent response to temperature and pressure. Structure determination by single-crystal X-ray diffraction at 200 and 100 K allows us to understand their response as a function of temperature. Applying hydrostatic/uniaxial pressure or grinding also generates significant variations in their emission. The high structural flexibility of the Cu(I)-I chain is significantly linked to the corresponding alterations in structure. Remarkably, pressure can increase the conductivity by up to 3 orders of magnitude. Variations in resistivity are consistent with changes in the band gap energy. The experimental results are in agreement with the DFT calculations. These properties may allow the use of these CPs as optical pressure or temperature sensors. In addition, their behavior as a heterogeneous photocatalyst of persistent organic dyes has also been investigated.

## Abstract 2

A mild, low-cost, solvent-free, one-step synthesis of smart 2D coordination polymers based on Cu(I)-I double chains with photocatalytic activity and remarkable luminescence and conductivity response to pressure and temperature is shown. Pressures and low temperatures induce changes in the cluster-core distances, producing drastic increases and variations in their emission. Additionally, pressure, either uniaxial or hydrostatic, reduces the band gap, increasing their electrical conductivity.

## Introduction

Coordination polymers (CPs) have received increasing attention over the past decades, favoring their rapid development in chemistry and materials science. The vast array of physicochemical properties and architectures they exhibit is the reason behind this. The great versatility of these compounds is achieved thanks to the proper selection of their different building blocks: metal ions and organic ligands joining through a self-assembly process.^1,2^

One of the main assets of CPs is the dynamic nature of their bonds and their structural flexibility. These characteristics are used to generate multifunctional materials that respond to multiple physical or chemical stimuli, producing a change in their properties. Accordingly, they can have interesting applications as sensors.^3^ Moreover, studies have been carried out on the electrical behavior of CPs both in bulk and in nanomaterials.^4,5^ One of the main goals in this context has been to exploit these materials in electronic devices, with a high degree of functionality, absent in common semiconductors or metals.^6,7^

An interesting CP family, due to its optoelectronic properties, is Cu(I)-halogen CPs, which are more affordable compared to other luminescent CPs based on noble metals or lanthanides.^8^ In particular, extensive research has been conducted on Cu(I)-I double chain-based coordination polymers due to their remarkable structural flexibility as well as their noteworthy characteristics in luminescence and semiconducting behavior.^9−12^ These CPs exhibit luminescence responding to pressure, temperature, the presence of gases, etc.^13,14^ As a result, they are well-suited substances for sensors that are both thermochromic and mechanochromic simultaneously^15^ with various industrial applications, such as aerospace, nuclear, food, packaging, and medicine.^16^

On the other hand, advances in experimental techniques in recent years are opening new perspectives in the production of functional materials under more extreme conditions of pressure or temperature.^17−21^ Indeed, previous studies of this type of Cu(I)-I CPs have correlated structure and photoluminescence (PL) in response to hydrostatic pressure or grinding.^14^

However, as far as our understanding goes, there has been limited exploration of the optical and electrical properties of flexible two-dimensional (2D) systems when subjected to hydrostatic pressure, with only a few examples observed in the context of porous three-dimensional (3D) CPs.^22,23^ Solid-state conductivity can be regulated by the application of high pressures, which leads to modifications in lattice parameters, electron–lattice interactions, and band gap. This makes studies on this subject quite intriguing.

In this work, the use of an N-donor ligand derived from pyrazine (2-fluoropyrazine; hereafter referred to as Fpyz) allows generating two almost identical two-dimensional (2D) CPs of chemical formula [Cu_2_I_2_(FPyz)]_*n*_ (**CP1** and **CP1’**) with electrical and luminescent responses to pressure and temperature. The modification of cluster-core distances and angles explains their behavior. Furthermore, the plentiful presence of metal nodes contributes to the emergence of semiconducting characteristics in certain CPs upon exposure to ultraviolet (UV) light or sunlight. This interesting characteristic encourages us to investigate its applicability as possible heterogeneous photocatalysts for the degradation of persistent dyes in water.

## Experimental Section

All reagents and solvents obtained were utilized as received without additional purification. Copper(I) iodide (CuI, 99%) and 2-fluoropyrazine of at least 97% purity were procured from Sigma Aldrich. Acetonitrile (CH_3_CN) with high-performance liquid chromatography (HPLC)-grade purity was sourced from Scharlau.

Data from the equipment used to obtain infrared spectra, elemental analysis, X-ray powder diffraction, thermogravimetric analysis, diffuse reflectance, photoluminescence, high-pressure luminescence, scanning electron microscopy, electrical conductivity, adsorption coefficient, high-pressure transport measurements, photocatalytic studies, and theoretical methods are found in Section 1 of the Supporting Information.

Single crystal X-ray diffraction (SC-XRD) measurements were conducted using a Bruker Kappa Apex II diffractometer equipped with a cryostat suitable for low-temperature or inert atmosphere collections. The diffraction data were collected at ambient pressure at both 200 and 100 K utilizing Mo Kα radiation that was monochromatized with graphite (λ = 0.7107 Å) at 50 kV and 30 mA. The crystal structures were determined using a dual-space algorithm implemented in the SHELXT program followed by refinement against F^2^ using the SHELXL program through full-matrix least-squares refinement. The cell parameters were determined and refined by fitting all reflections with a least-squares approach. Additionally, a semiempirical absorption correction (SADABS) was applied in the analysis.

For high-pressure measurements, we employed a Rigaku SuperNOVA diffractometer with microfocus X-ray utilizing Mo Kα radiation. To facilitate these measurements, a Bragg-Mini diamond anvil cell (DAC) from Almax-EasyLab was utilized, featuring an 85° opening angle and diamond anvils with culets measuring 500 μm in diameter. The DAC was equipped with a stainless gasket that had a 200 μm diameter and 75 μm depth hole. To minimize deviatoric stresses and ensure accurate bulk modulus values, a methanol–ethanol mixture (4:1) was utilized as the pressure-transmitting medium throughout the experiments. This pressure-transmitting medium was employed across the range of pressures applied to maintain hydrostatic conditions.

Sample **CP1’** (refer to the description below) was positioned on one of the diamond anvils (the diffracted side), accompanied by a small ruby sphere serving as a pressure sensor. For each pressure point, the crystal structure was refined using the SHELXL program based on F^2^ values via full-matrix least-squares refinement utilizing previous results as the starting point. Given the limited opening angle of the DAC, only approximately 45–50% of the complete data set’s reflections under ambient conditions could be collected. Consequently, structure refinements were performed with isotropic displacement parameters for all atoms, except for the heavy atoms (Cu and I), which were refined with anisotropic displacement parameters except when their respective thermal factors were physically inconsistent. Hydrogen atoms were incorporated into the final refinement procedure following the same approach as for ambient conditions, and no restraints were applied.^24^

Crystallographic data for the structures reported in this contribution have been deposited with the Cambridge Crystallographic Data Centre as supplementary publication 2240072–2240083. Copies of the data can be obtained free of charge on application to the CCDC, Cambridge, U.K. (http://www.ccdc.cam.ac.uk/).

### Synthetic Procedures

#### Hydrothermal Synthesis of [Cu_2_I_2_(Fpyz)]_*n*_ (**CP1**) Single Crystals (*P*-1 Polymorph)

CuI (38 mg, 0.2 mmol), 0.7 g of KI, and the ligand 2-fluoropyrazine (18 μL, 0.2 mmol) were dissolved in 5 mL of water. The solution was heated to 120 °C in 2 h, left for 48 h at 120 °C, and slowly cooled down to RT for another 24 h (3.8 °C/h). After that time, floating orange crystalline fibers appeared and were separated manually. The crystals were washed with CH_3_CN (2 × 2 mL) and dried on air. Yield was 20% (19 mg) based on Cu. Elemental analysis calculated (%) for C_4_H_4_FCu_2_I_2_N_2_ was C 10.03, H 0.63, and N 5.85; experimental results were as follows: C 10.00, H 0.72, and N 5.83. Selected peaks IR (ATR) (cm^–1^) = 3071 (w), 1593 (s), 1455 (m), 1405 (s), 1285 (m), 1151 (m), 1030 (s), and 850 (s) (Figure S6).

#### Solvent-Free Mechanical Synthesis of [Cu_2_I_2_(PyzF)]_*n*_ (**CP1**) Polycrystals (*P*-1 Polymorph)

**Figure 1:**
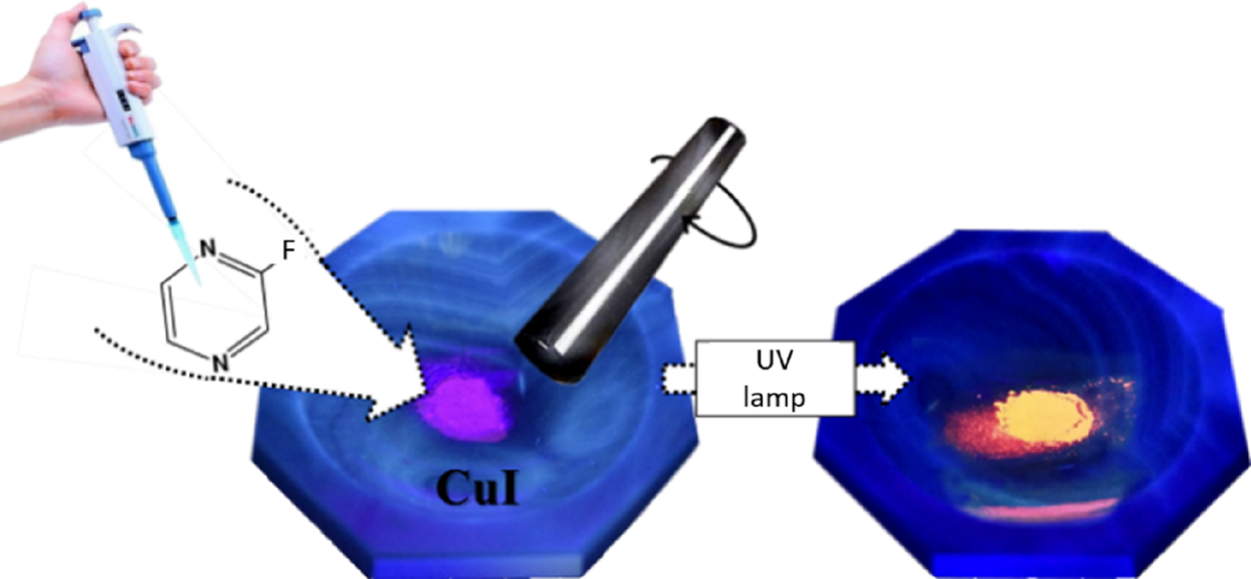
Synthesis of [Cu_2_I_2_(FPyz)]_*n*_ (**CP1**) by solvent-free methodology conducted using mechanical grinding in an agate mortar under the illumination of a UV lamp (λ = 365 nm). This process induces a change in the color of the photoluminescence from purple to orange.

The synthesis was carried out mechanically in an agate mortar under a UV lamp (λ = 365 nm) to verify the formation of the product (Figure S1). CuI (190 mg, 1 mmol) and 2-fluoropyrazine (82 μL, 1 mmol) were added to the mortar at room temperature. The initial violet mixture was ground for 2 min, giving an orange (**CP1**) compound (Figure. 1 and Figure S1). The solid was washed with acetonitrile (2 × 5 mL) and separated by centrifugation for 10 min at 6000 rpm. Finally, the coordination polymer was dried for 15 min under a vacuum.

#### The Powder X-ray Diffractogram Corroborates the Purity of the Phase (Figure S7a)

**CP1**: yield: 74% (215 mg) based on Cu. Elemental analysis calculated (%) for C_4_H_4_FCu_2_I_2_N_2_ showed the following: C 10.03, H 0.63, and N 5.85; experimental results were as follows: C 10.00, H 0.80, and N 5.86. Selected peaks. IR (ATR) (cm^–1^) = 3078 (w), 3020 (w), 2918 (w), 2850 (w), 1592 (m), 1526 (s), 1456 (m), 1405 (s), 1284 (m), 1171 (m), 1150 (m), 1150 (m), 1065 (m), 1030 (s), and 850 (s) (Figure S6).

Additionally, **CP1** is partially soluble in acetonitrile (orange color solution). For this reason, in the washing solution at room temperature for about 24 h, large crystals corresponding to the polymorph *P*2_1_ (**CP1’**) were obtained.

#### Synthesis of [Cu_2_I_2_(PyzF)]_*n*_ (**CP1’**) (*P*2_1_ Polymorph) in Acetonitrile

An ultrasonic bath (25 °C, 60% power, 40 KHz) was used to dissolve the mixture between 2-fluoropyrazine (82 μL, 1 mmol) and CuI (190 mg, 1 mmol) in 5 mL of acetonitrile. An orange (**CP1’**) precipitate was formed after sonication for 10 min (Figure S2). The solid was filtered off, washed with acetonitrile (6 mL), and dried in a vacuum. Additionally, by slow evaporation at room temperature from mother liquors, a greater amount of **CP1’** in the form of single crystals was also obtained.

**CP1’**: yield: 25% (55 mg) based on Cu. Elemental analysis calculated (%) the following for C_4_H_4_FCu_2_I_2_N_2_: C 10.03, H 0.63, and N 5.85; experimental results were as follows: C 9.95, H 0.82, and N 5.78. Selected peaks IR (ATR): n (cm^–1^) = 3078 (w), 3020 (w), 2918 (w), 2851 (w), 1593 (m), 1526 (s), 1456 (m), 1406 (s), 1284 (m), 1171 (m), 1150 (m) 1065 (m), 1030 (s), and 850 (s). The powder X-ray diffractogram corroborates the purity of the phase (Figure S7b).

## Results and Discussion

**Scheme 1:**
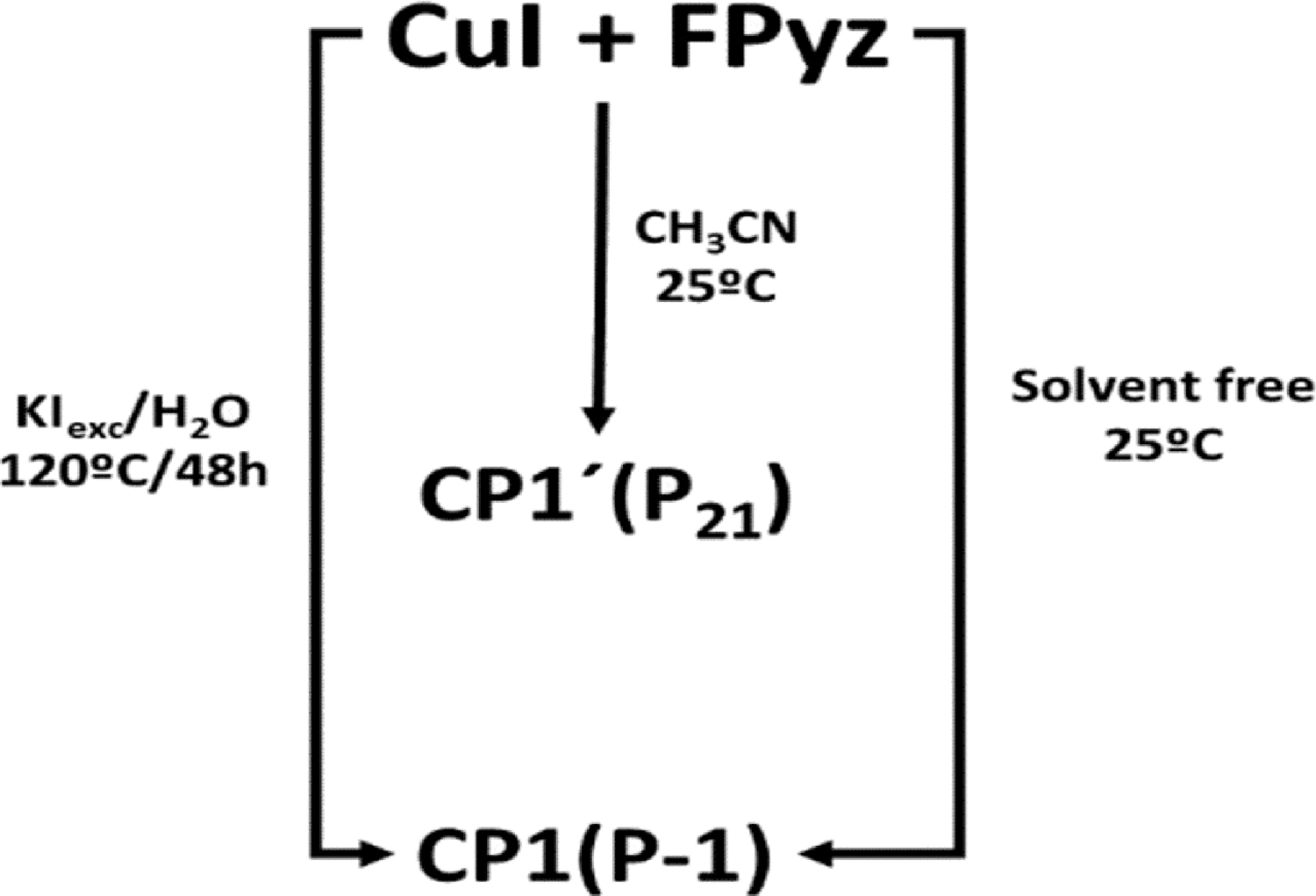
Synthetic Routes to get **CP1** and **CP1’** Polymorphs (2-Fluoropyrazine (Fpyz))

As discussed later, the coordination polymers **CP1** and **CP1’** are polymorphs (space groups *P*-1 and *P*2_1_, respectively) and can be isolated with better yield by different synthesis routes (Scheme 1).

Thus, the solution of CuI in excess of KI using water as solvent with stoichiometric amounts of FPyz allows us to carry out a hydrothermal synthesis at 120 °C for 48 h. The slow cooling of the reaction allows us to obtain crystals corresponding to **CP1** (*P*-1 polymorph) suitable for SC-XRD (Scheme 1). Furthermore, the identical polymorph can be predominantly produced through a solvent-free mechanical reaction visually monitored under UV light, facilitated by the strong photoluminescence of the resulting CP and the absence of emission from the chosen liquid organic ligand. As a result, the original purple emission of CuI from the initial mixture evolves to a vibrant orange emission in **CP1** (Scheme 1, Figure 1, and Figure S1).

Additionally, it has already been reported^37^ that the dissolution of some [CuI(L)]_*n*_ (L = organic ligands) CPs in acetonitrile entails the rupture of the CP into its different constituents, which are reorganized during the slow evaporation of the solvent. By employing this principle and leveraging the limited solubility of **CP1** in acetonitrile, it becomes feasible to conduct the synthesis of **CP1’** (*P2*_1_) through the process of crystallization at room temperature from the remaining solution.

The formation of these two polymorphs exhibiting slight structural differences can be tentatively attributed to the solvent environment during the synthesis process. The intermolecular interactions between CH_3_CN and the coordination framework presumably induce the formation of **CP1’**. As expected, both polymorphs have similar properties; both are stable in air, are insoluble in most common organic solvents (except CH_3_CN), and have very similar X-ray powder diffractograms (Figure S7a,b) and thermal stability (sample degradation above 180 °C, Figure S9). Moreover, both compounds have the same IR (Figure S6) displaying the characteristic IR absorption spectra of the 2-fluoropyrazine ligand that undergo a shift in the CP. The shift of the strongest ν C–N band from 1012 cm^–1^ in the ligand to 1030 cm^–1^ in **CP1** and the ν C–F band from 1262 to 1285 cm^–1^ is indicative of its coordination with the metal center. The intermediate bands between 1060 and 1170 cm^–1^ are assigned to the −CH_2_– vibrations.^25^

Indeed, as we will discuss later, they have very similar photoluminescence spectra.

### Crystal Structures of [Cu_2_I_2_(FPyz)]_*n*_ (**CP1** and **CP1’**)

**Figure 2:**
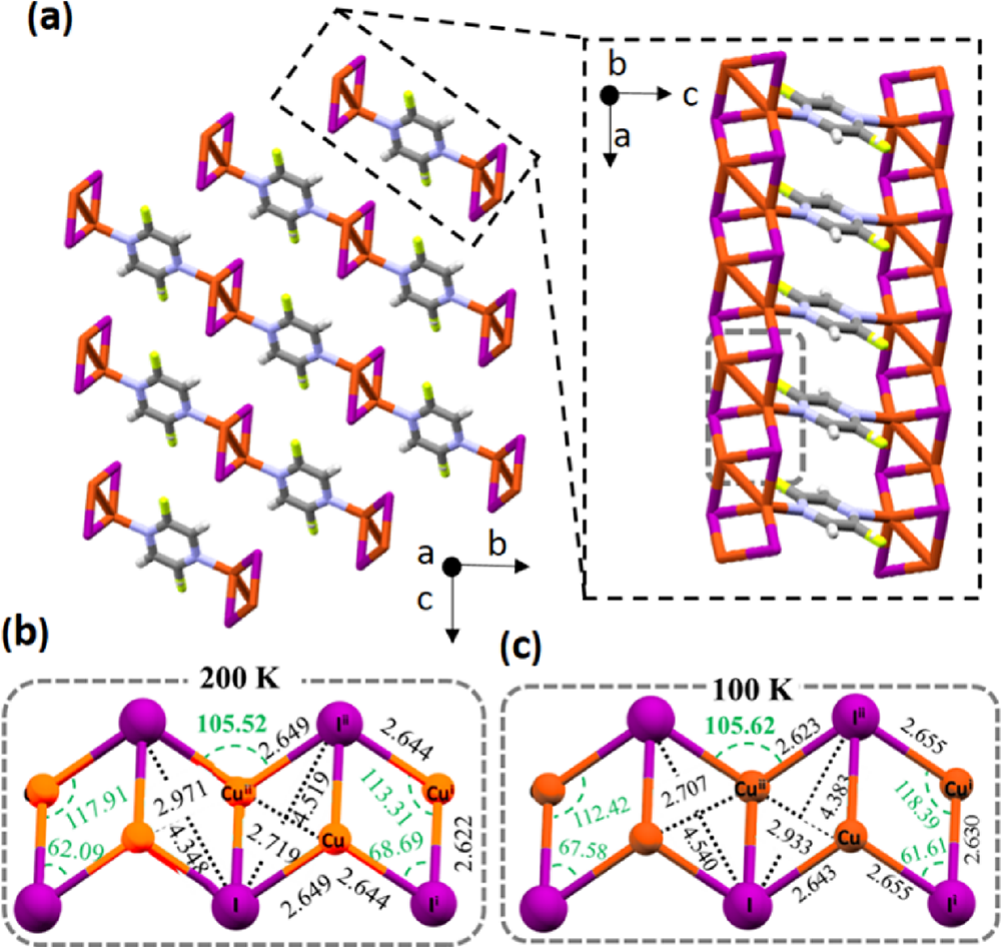
Mercury CCDC representation of 2D [Cu_2_I_2_(FPyz)]_*n*_ (**CP1**) packing. Chain expansion across the *a* axis for **CP1** (a). Zoom of the Cu-I chains for **CP1** at 200 K (b) and at 100 K (c) with the angle measurements (green) and distances (in Å) (black).

Table S1 contains the geometrical parameters, whereas Table S2, Figure 2, and Figures S3 and S4 show the distances and angles for **CP1**, respectively, which were determined by single-crystal X-ray diffraction at 200 and 100 K.

Additionally, the structure of its polymorph **CP1’** was also solved at 100 K (Tables S1 and S2, Figures S3–S5). The two-dimensional arrangement of **CP1** and **CP1’** showcases a double-stranded ladder motif, wherein the Cu(I) centers are interconnected by μ_3_-I bridges. This Cu(I)-I double chain represents a distinctive coordination mode commonly observed in these kinds of CPs. The copper(I) atom is bonded to one nitrogen atom of the 2-fluoropyrazine ligand with a trigonal pyramid local coordination, giving a 2:1 CuI/L ratio and neutral charge (Figure 2 and Figure S3). The most relevant differences between both polymorphs consist of the orientation of the fluorine atom (Figures S4 and S5). The comparison of distances and angles at 100 K for both polymorphs can be found in Table S2. Cu–N bond distances are 2.074 and 2.094 Å for **CP1** at 200 and 100 K, respectively, and 2.099 Å for **CP1’** (100 K), in agreement with the values found in the CSD database for similar chains based on Cu(I)-I (Table S2).^10^

Additionally, both compounds show an asymmetric core with Cu···Cu distances of 2.719 and 2.971 Å at 200 K (**CP1**), 2.707 and 2.933 Å at 100 K (**CP1**), and 2.888 and 2.729 Å at 100 K (**CP1’**), respectively (Table S2). All the measured distances closely approximate the sum of the van der Waals radii, which is typically around 2.8 Å. This specific distance range is significant as it indicates the minimum proximity required for noticeable attractive interactions to occur.^26^ Their supramolecular interactions are van der Waals type, corresponding to a distance above 3.6 Å. Crystallization proceeds in the form of a thicker plate thanks to the two Cu-I double chains per ligand (Figure 2 and Figures S3 and S4).

### Structural Changes of the **CP1’** Crystal Subjected to Hydrostatic Pressure (HP)

The **CP1** (*P*-1) crystals obtained by hydrothermal synthesis are sufficiently small to be subjected to a pressure study. However, the quality of the crystals obtained by recrystallization in acetonitrile (**CP1’**) allows its use to study and correlate structural variations under hydrostatic pressure with variations in luminescence and conductivity (see below). A **CP1’** single crystal was placed into a DAC cell together with an ME 4:1 liquid as a pressure transmission medium and underwent a gradual increase of the pressure from 0 to 4.5 GPa.^40^

The results obtained from this experiment facilitate the calculation of the isothermal equation of state and the bulk modulus (*V*_0_ = 444.39(7) Å^3^, *K*_0_ = 11.6(5) GPa, and *K*’_0_ = 9.2(8)) (see Section S5 of the Supplementary Information, Table S3, and Figures S10–S12). These findings elucidate the degree of material compressibility, which falls within the range typically observed for organometallic compounds and exhibits similarities to other recently published Cu(I)-I CPs.^14,27^ The fitting procedure was performed utilizing the EosFit7-GUI program employing the BM3 equation of state. As observed, this compound has an anisotropic behavior in its compressibility, with the *b* axis being much softer than the other two unit-cell axes. Furthermore, the structural changes induced by an increase in pressure are notable, as evidenced by significant variations in certain distances and angles (Table 1; Figure S12). Notably, the most significant changes occur in the (diagonal) Cu–Cu distances, which decrease by up to 10% as the pressure increases, resulting in a slippage in the stair-like structure. This can also be observed in the variation of I–Cu–I (trans) bond angles along the chain (Figures S11 and S13).

**Table 1:** Representative Variations in the Cu···Cu Distances and I–Cu–I and Cu–I–Cu Angles for Compound **CP**1**’** Subjected to Pressures of 0 and 4.47 GPa, Respectively, and the Difference between Them (Δ)

bonds and angles	298 K, 0 GPa	298 K, 4.47 GPa	Δ
Cu···Cu^i^	2.754(3)	2.634(7)	–0.120 (4.4%)
Cu···Cu^ii^	2.937(3)	2.639(8)	–0.298(10.1%)
Cu–I	2.635(2)	2.605(7)	–0.030 (1.1%)
Cu–I^i^	2.658(2)	2.578(7)	–0.080 (3.0%)
Cu–I^ii^	2.632(3)	2.589(9)	–0.043 (1.6%)
Cu–N	2.07(1)	2.04(3)	–0.03 (1.4%)
Cu–I–Cu^ii^	67.72	61.0	–6.72 (9.9%)
Cu–I^ii^–Cu^ii^	67.70	61.0	–6.70 (9.9%)
Cu–I^i^–Cu^i^	117.12	118.6	1.48 (1.3%)
Cu–I^ii^–Cu^i^	62.65	61.2	–1.45 (2.3%)
Cui–I^ii^–Cu^ii^	106.31	105.8	–0.51 (0.5%)
I^i^–Cu–I^ii^	117.55	118.9	1.35 (1.1%)
I^ii^–Cu–I	112.47	118.9	6.43 (5.7%)
I–Cu–I^i^	106.71	105.8	–0.91 (0.9%)
I–Cu^ii^–I^ii^	112.10	118.9	6.80 (6.1%)
I^i^–Cu^i^–I^ii^	117.12	118.6	1.48(1.26%)

### Optical Properties

#### Temperature-Dependent Photoluminescence

**Figure 3:**
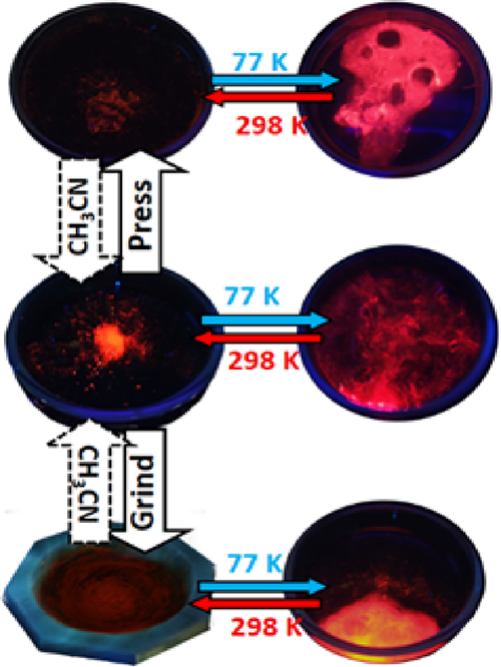
PL image of **CP1** under different conditions. The middle image displays the emission at 298 and 77 K under UV light (λ = 365 nm), whereas the top image shows the emission after pressure of 5.5 GPa for 10 min. The bottom image illustrates the emission resulting from grinding the sample for 10 min. In both cases, application of pressure or grinding, respectively, a notable recovery of the PL of CP can be observed upon the addition of acetonitrile to the samples.

A qualitative test at 298 K under UV irradiation (λ_exc_ = 365 nm) shows that both polymorphs present orange emission. The same test also shows a change in the emission when the temperature decreases to 77 K (Figure 3 and Figure S14).

**Figure 4:**
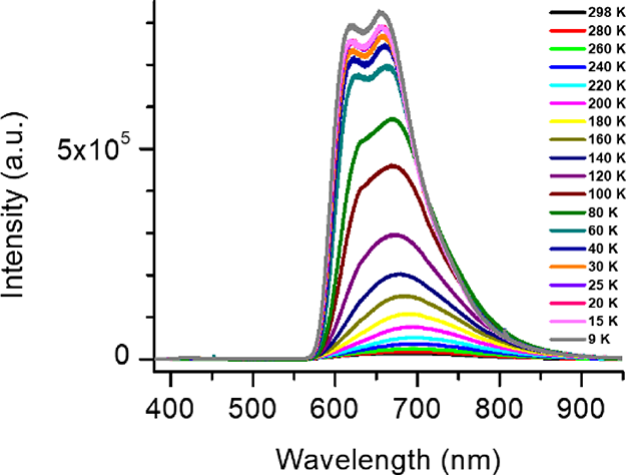
PL spectra of compound **CP1** as a function of temperature (λ_exc_ = 355 nm). The spectra demonstrate a strong increase of intensity, an initial red shift followed by a blue shift, and the appearance of fine structure with decreasing temperature (see also Figure S14).

The photoluminescence spectra of **CP1** (*P*-1), obtained by a solvent free mechanical reaction, were monitored as a function of temperature (Figure 4). At 280 K, the PL spectrum of **CP1** (Figure S15) shows a broad emission band centered at 665 nm, which narrows and red-shifts toward 683 nm as the temperature decreases down to 220 K, followed by a blue shift and the appearance of two peaks at 618 and 656 nm as the temperature reaches 9 K. With decreasing temperature, its luminescence is dramatically enhanced, resulting in an enhancement of the peak PL intensity by a factor of 57 at 9 K relative to 298 K (enhancement in PL area by a factor of 37). It is noteworthy that the properties of the compound exhibit a significant degree of reversibility, where the gradual recovery of its initial characteristics can be observed upon heating from 9 to 298 K, indicating the intrinsic temperature sensitivity of this luminescent material.

At the same time, the PL decay curves become nonexponential at low temperatures, and the intensity-averaged lifetimes increase almost exponentially by a factor ≈600 upon lowering the temperature from 300 to9 K (Figure S16). This indicates efficient nonradiative decay at room temperature. The wavelength independence of the decays indicates that only one emitting state contributes to the emission.

**Figure 5:**
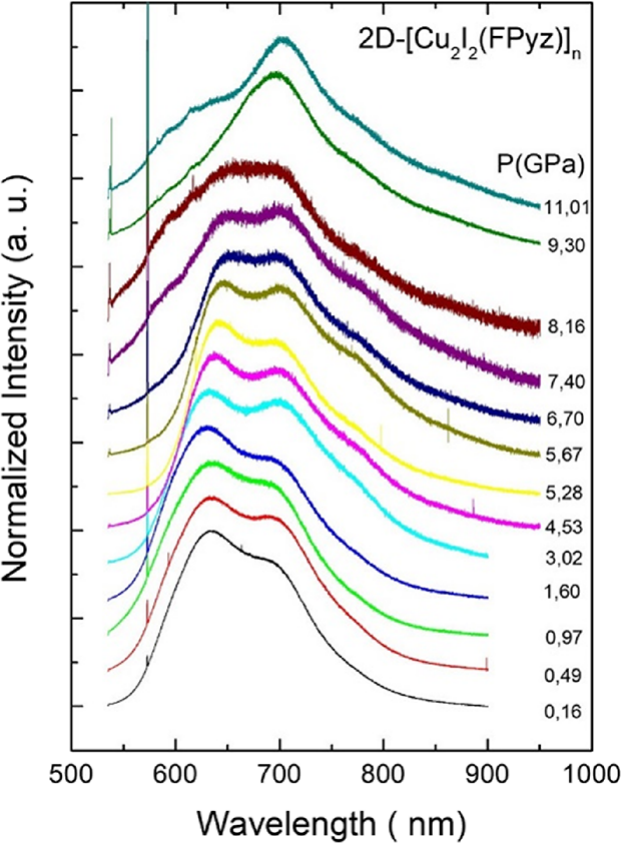
Normalized room temperature PL spectra of **CP1** under hydrostatic conditions excited with a diode laser at 532 nm.

In this compound, the highest occupied molecular orbital (HOMO) can be described as a combination of the 3d orbitals of Cu^+^ and the 4p orbitals of I,^28,29^ whereas the first unoccupied molecular orbital (LUMO) primarily corresponds to π* orbitals of the corresponding pyrazine ligands. Consequently, the observed optical transitions are attributed to σ-donor−π-acceptor charge transfer (CT) transitions facilitated by the presence of conjugated bonds. The excitation energy enables electrons to be promoted from the occupied CuI orbitals to the vacant antibonding ligand orbitals, resulting in a d → π* MLCT-type transition, located within the UV–visible range. Previous studies involving similar ligands^30−32^ have provided evidence that the emission arises from a mixed charge transfer process involving iodide and copper to the ligand [(Cu + I) LCT] that is facilitated by the short distances between Cu–I and Cu–N. Nevertheless, even at room temperature, **CP1** exhibits Cu···Cu distances that are shorter than the sum of the van der Waals radii (2.8 Å) or at least half of that distance (2.971(2) Å, refer to Table S2). This indicates the presence of cuprophilic interactions, which are consistent with ^3^CC and ^3^ICuCT-type transitions involving a CuI core. Furthermore, the asymmetric nature of the PL band implies the involvement of multiple states in the emission process.^11^ The emission intensity of CPs is significantly increased at a lower temperature of 9 K (Figure 4), which can be attributed tentatively to the spring-like behavior of the Cu(I)-I chains. This behavior is due to the shortening of Cu···Cu and Cu–I distances and contraction of I···Cu···I angles at lower temperatures as evidenced by the crystal structures obtained at 200 and 100 K through SC-XRD. As a result, the material becomes more rigid, which inhibits nonradiative relaxation processes. It is noteworthy that this behavior is reversible, as the material gradually recovers its initial properties, including low PL quantum yield, upon heating from 9 to 298 K, demonstrating its inherent temperature dependence (Figure 2 and Table S2).^16,26,33^ Therefore, there is evidence that the nonradiative decay processes and, hence, PL intensity are governed by the Cu···Cu distances. Even at 9 K, [(I + Cu) LCT] transfer still seems relevant, as the shift occurs toward higher energies. Furthermore, the optical band gap of **CP1** has been determined to be 2.1 eV, consistent with this argument (Figures S17 and S18). Because of the close correlation between the structure and emission in the solid state, mechanical stresses at high pressure alter significantly the arrangement of Cu–I chains, affecting the inter/intramolecular interactions with similar effects on the emission properties observed with temperature. Thus, studies on mechanoluminescence were conducted by applying hydrostatic pressure. As we have mentioned above, in these Cu(I)-I CPs, the emission is a consequence of transitions of mixed ^3^(Cu + I)LCT charge transfer states (referred to as the high-energy or HE band) and CuI cluster-core (^3^CC) states including the cuprophilic interaction of metal-cluster-centered d^10^ → d^9^s^1^ and iodide-to-copper ^3^ICuCT charge transfer (referred to as the low-energy or LE band). In **CP1**, both HE and LE-type bands seem to coexist. On the basis of the structural data obtained from the high-pressure X-ray diffraction experiments, we can gain insights into the luminescent properties of **CP1**. Specifically, the bond distances of Cu–N, Cu···Cu, and the CuI cluster-core are known to be closely related to the luminescence properties of **CP1**. Accordingly, changes in the Cu–N, Cu···Cu, and Cu–I distances with pressure can be correlated with changes in HE and LE band contributions in the luminescence spectra. The separated integrated intensities were evaluated considering the whole spectra. High-pressure luminescence experiments were recorded at room temperature exciting at 532 nm. Up to three sub-bands were observed in the emission spectra of **CP1**, at ambient conditions centered at 634 nm (LE1), 694.6 nm (LE2), and 701.5 nm (LE3), respectively (Figure 5). The evolution of both components was monitored, and minor changes in line-shape were observed up to 4.0 GPa. Beyond this pressure, the LE1 shows a red shift, and LE2 and LE3 remain almost unchanged in position until the latter disappeared. On the other hand, at 8.1 GPa, there is an increase of the overlapping between them, and above this pressure, the relative intensity of LE1 strongly diminishes, with only the LE2 contribution remaining. The integrated intensity shows a rapid decrease of six times below 2 GPa at the initial states and then gradually decreases down to 98% at 11 GPa from its initial values at ambient pressure. Decompressing the sample to ambient pressure impacts the recovery of the initial luminescence features.

**Figure 6:**
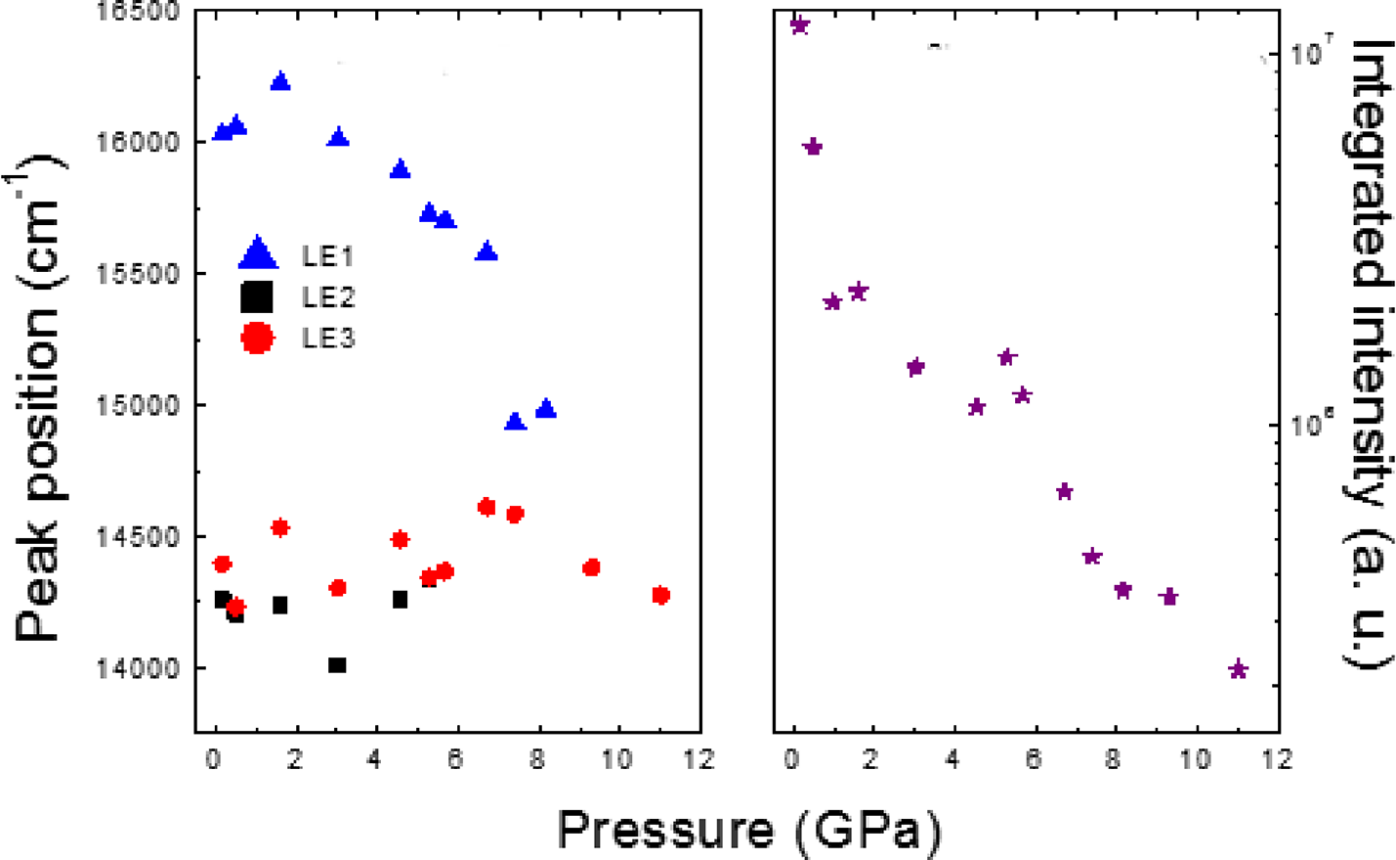
Shifts in peak positions of the emission under 532 nm excitation and analysis of the corresponding integrated intensities for **CP1** as a function of pressure.

For compound **CP1**, the Cu···Cu, Cu–I, and Cu–N distances up to 4.5 GPa were recorded (Table 1). Regarding the former, two different bond distances were defined for a couple of Cu ions, the Cu···Cu^i^ and Cu···Cu^ii^ ones, obtained from X ray diffraction at ambient conditions at 2.937 and 2.754 Å, respectively. The shortening with pressure reaches 10 and 4.2%, respectively, in the ambient to 4.5 GPa interval. At first sight, there is a large difference in the rate of shortening for both bond distances, but considering that the cuprophilic interaction becomes non-negligible at distances less than 2.8 Å, Cu···Cu^i^ interactions would start to influence the emission around 1.5 GPa, when the distance is around 2.779 Å. This observation translates into a change of around 5.2% for the pressure interval from 1.5 to 4.5 GPa, which is near the Cu···Cu^ii^ one. On the other hand, in the case of the Cu–N and Cu–I distances, both show slight shortenings, mainly in the interval from ambient to 3.8 GPa, reaching less than 2%. It is important to note that the pressure achieved for the luminescence experiment was around 11 GPa and is precisely above 4.5 GPa where the major variations of the spectra were observed. It is known that the shortening of the Cu···Cu distances induces a red shift in the luminescence band. Thus, we identify the LE1 to transitions with cuprophilic interaction of metal-cluster-centered d^10^ → d^9^s^1^ (^3^CC) and LE2 and LE3 with a mixing of ^3^(Cu + I)LCT and ^3^ICuCT (Figure 6). It is worth mentioning the role played by the pressure in the nonradiative processes that leads to an almost total quenching of the emission for pressure higher than 10 GPa.

#### Mechanoluminescence Studies by Grinding and Uniaxial Hydraulic Pressure

**Figure 7:**
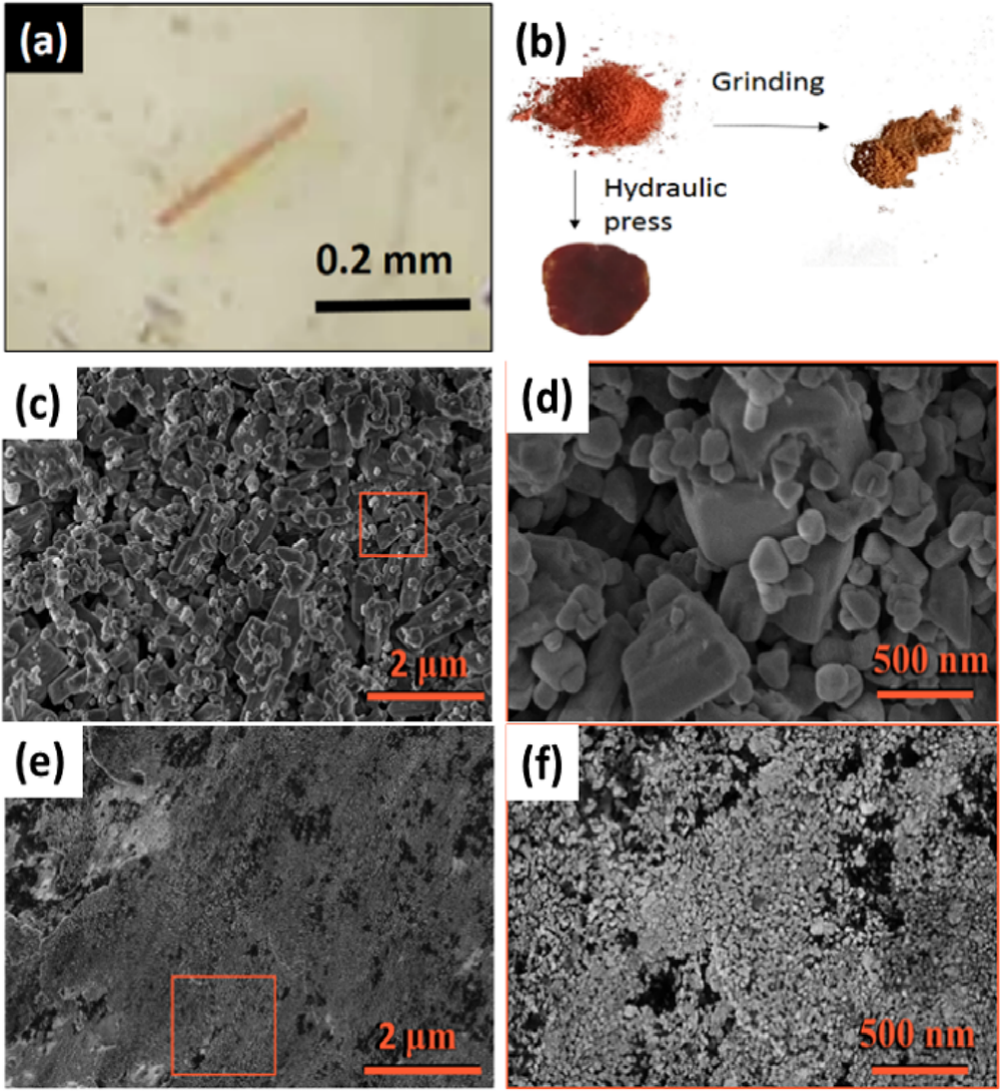
Bright-field microscope image of a single crystal **CP1** (space group *P*-1) (a). Visual image of polycrystalline powder and after having been subjected under pressure (b). SEM images of **CP1** obtained after solvent-free synthesis by grinding (2 min) (c) and zoom into the indicated area at higher magnification (d). SEM images of **CP1** after 10 min of hydraulic press at 5.5 GPa (e) and zoom into the indicated area at higher magnification (f) (for **CP1’**, see Figure S21).

**Figure 8:**
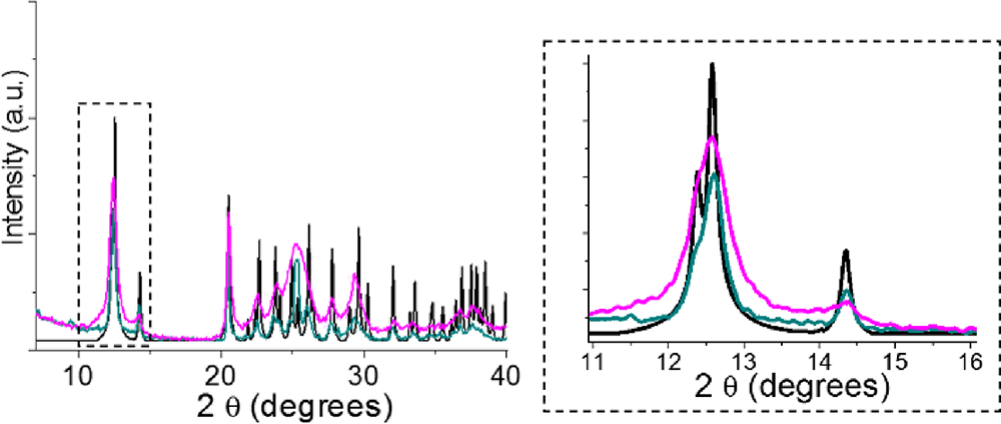
X-ray powder diffractogram of **CP1** (black line), **CP1** after 10 min of grinding (pink line), and **CP1** after 10 min of hydraulic press at 5.5 GPa (green line). A scale-expanded diffractogram is shown on the right-hand side.

As previously published,^34,35,9^ grinding the material can cause crushing of the compound, with the generation of granules and defects, where the compound undergoes variations in emission intensity and/or wavelength. Accordingly, the pressure-induced changes in luminescence were monitored in **CP1** at RT upon screening powders that were subjected to different grinding times (Figure S19) and at different hydraulic pressures (Figure 3). The studies show that mechanical action causes a decrease in the emission intensity (Figure S20). This variation is attributed to the mechanical effort made on the sample that, although does not produce a structural change (Figure 8), reduces the size of the crystals generating in turn an increase in the number of defects. This has been observed by scanning electron microscopy (SEM), revealing lengths of the starting needle-shaped single crystals of 0.2 mm (Figure 7a and Figure S22) and grains of 147 ± 100 nm in the ground material (Figure 7c,d and Figure S21). Furthermore, when **CP1** is subjected to a uniaxial pressure of 5.5 GPa by a hydraulic press for 10 min, a reduction in grain size also occurs (Figure 7e,f and Figure S21). In both cases, grinding or hydraulic pressing does not cause amorphization or phase change, as we can see in their X-ray powder diffractograms obtained after 10 min (pink and green line, respectively, in Figure 8). However, they depict a lower definition and inhomogeneous broadening, particularly upon grinding. These changes are associated with a small loss of crystallinity, the presence of strain, and a reduction of the grain size.^36−38^

Dissolving the ground and hydraulically pressed **CP1** (10 min) powders in acetonitrile enables the recrystallization of **CP1** in the form of high-quality micrometric single crystals and the recovery of the original emission, which confirms that the reduction in size and the creation of defects are the main causes of the emission changes.

### Electronic Properties vs Pressure

There are several studies of the pressure effect on semiconductor materials. In the case of organic crystals, the most general concept is that the resistivity normally decreases with increasing pressure until this pressure produces irreversible changes in the structure. However, the application of uniaxial pressure to CPs produces an increase in resistivity attributed to the anisotropic structure of this type of materials and also to the formation of boundary grain defects along the pellet.^39−41^

The electrical conductivity of the compounds has been measured at 25 °C through the two-contact method. It should be noted that the crystals of **CP1’** are of better quality than those of its corresponding polymorph **CP1**. The measurement of the **CP1’** single crystals at 0 GPa shows a value of 3.1 × 10^–5^ S/cm (Table 2 and Figure S23b). However, when these crystals are subjected to a uniaxial pressure of 3.8 GPa, the conductivity increases by 3 orders of magnitude (Figure S23c). We have corroborated that the behavior of the **CP1** under the same conditions of pressure is similar (pellets at 3.8 GPa for 10 min, Table 2). We have also compared the behavior of **CP1** against uniaxial pressure starting both from single crystals and from polycrystals, finding that in both cases, despite the formation of grain boundaries, there is an increase in conductivity. This result seems to eliminate the possibility that the presence of CuI impurities could be the cause of the mentioned increase.

**Table 2:** Conductivity Data for **CP1** and **CP1’** as a Function of Pressure at 25 °C Obtained through the Two-Contact Method

	pressure (GPa)	conductivity (S/cm)
**CP1’** (single crystal)	0	3.1 × 10^–5^
**CP1’** (pellet) from single crystals	3.8	1.1 × 10^–2^
**CP1** (pellet) from single crystals	3.8	4.4 × 10^–1^
**CP1** (pellet) solvent free, 2 min grinding	3.8	1.1 × 10^–1^
**CP1** (Pellet) Solvent free, 2 min grinding	5.5	1.4 × 10^–1^

The changes in the conductivity are not attributed to any structural transformation because the X-ray powder diffractograms, as well as the IR, of both CPs after pressure do not show any structural change (Figure S24d). However, it can be related to an ordering of the crystal or a decrease in the band gap.

Next, we proceeded to study the variation of the resistivity of **CP1’** crystals under quasi-hydrostatic pressure.

**Figure 9:**
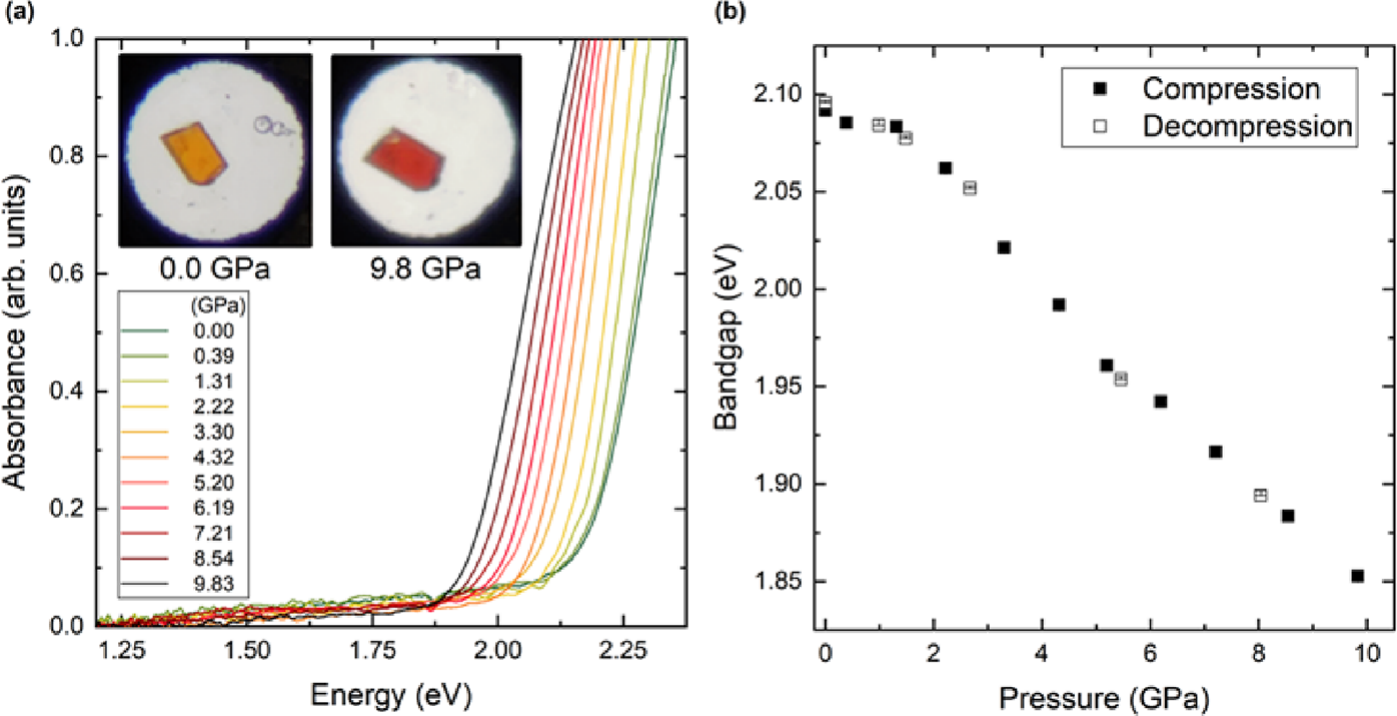
(a) Absorbance at different pressures measured in a **CP1’** single crystal. The pictures illustrate the color change of the crystal related to the decrease of the band gap energy. (b) Pressure dependence of the band gap energy measured under compression and decompression.

A single crystal of **CP1’** was introduced in a diamond-anvil cell. A selection of measured absorbance spectra is given in Figure 9a. There is a clear red shift of the fundamental absorption edge as pressure increases. This shift induces a color change in the sample from orange at ambient pressure to red at 9.8 GPa. The data acquired from this experiment enable the calculation of the changes in the band gap energy of the CP as a function of pressure, ranging from ambient pressure to 9.8 GPa (Figure 9b). The band gap energy is 2.1 eV at 0 GPa and decreases to 1.85 eV at 9.8 GPa.

The change induced by pressure in absorbance is reversible. The red shift of the band gap energy is fully consistent with results from our luminescence measurements. The origin of this phenomenon could be related with an enhancement of orbital hybridization induced by pressure, which will reduce the splitting between bonding and antibonding states near the Fermi level, thus reducing the gap between the top of the VB and the bottom of the CB.

**Figure 10:**
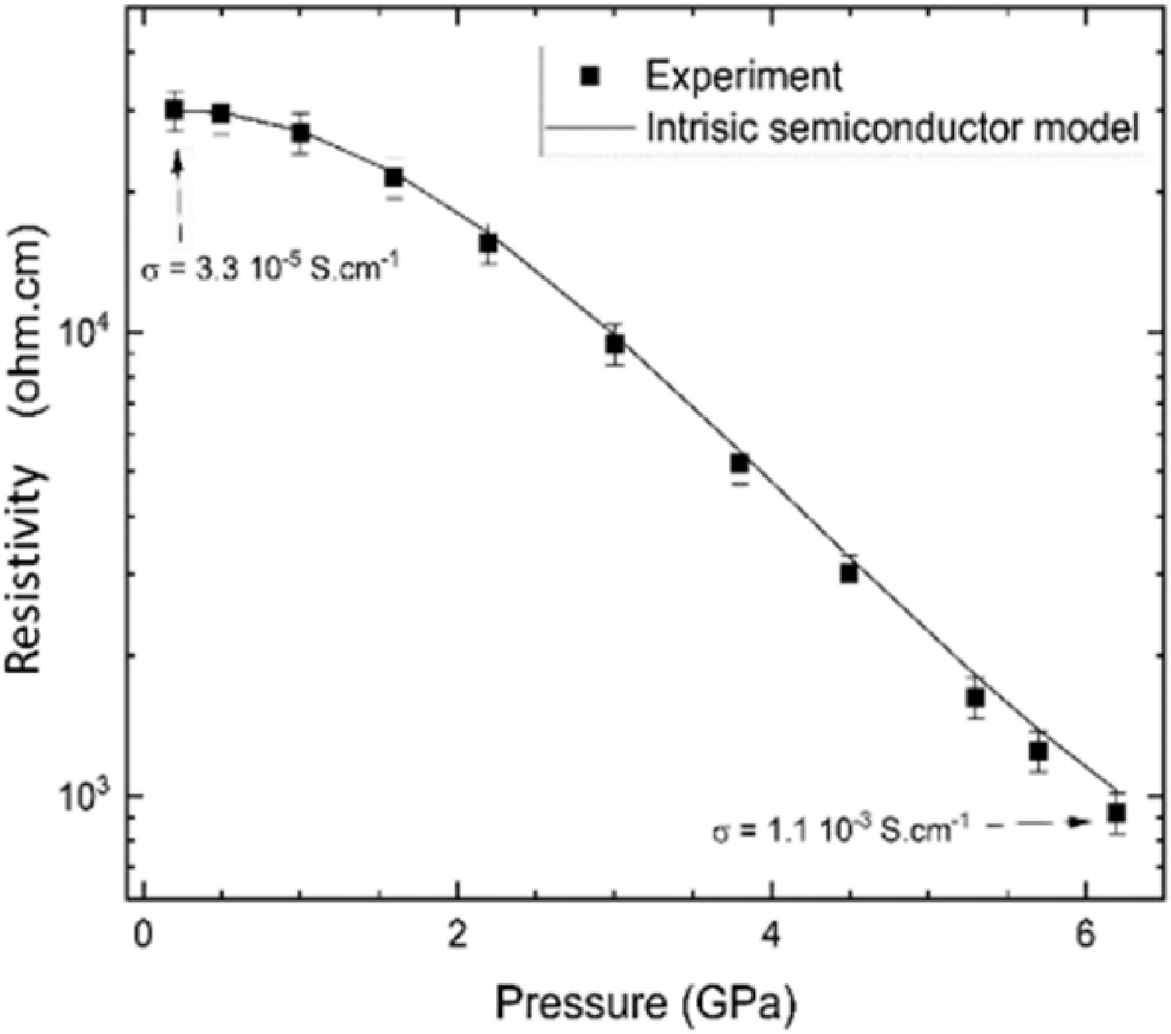
Pressure vs resistivity of the CP1**’** crystal investigated in Figure 9. The solid line is the calculated resistivity assuming the pressure dependence of the band gap reported in Figure 9b.

Now, we will discuss the results of in situ resistivity measurements under hydrostatic pressure depicted in Figure 10. As can be seen, the resistivity decreases under compression. The resistivity measured at 0 GPa is equivalent to a conductivity of 3.3 × 10^–5^ S/cm, which is compatible with the conductivity measured outside the DAC (Table 2). At 6 GPa, the resistivity corresponds to a conductivity of 1.1 × 10^–3^ S/cm, corresponding to an increase by a factor of around 30. The observed increase in conductivity in the in situ measurements is compatible with changes observed in the conductivity measured ex situ in pellets after pressurization. Thus, both methods give qualitatively similar results. The decrease of the resistivity can be correlated with the observed decrease of the band gap energy assuming that CP is an intrinsic semiconductor. The obtained pressure dependence for the resistivity assuming this hypothesis is plotted with a solid line in Figure 10. As can be seen, the solid line satisfactorily explains the experimental results. This means that the main factor behind the increase of conductivity is the decrease of the band gap energy. The consistency between resistivity and optical experiments indicates that the use of KBr as a pressure medium in resistivity experiments does not modify the behavior observed in the CPs up to 6 GPa using more hydrostatic pressure media. These results and conclusions have been corroborated by DFT calculations (see the discussion below).

#### DFT Calculations vs Pressure

To analyze the effect on the electronic properties of the compound of an externally applied hydrostatic pressure, we have computed the density of electronic states as a function of the energy (referred to as the Fermi energy) for the system at 0.0 and 3.8 GPa (Figure S25) The compound at these two pressures exhibits a narrow-gap semiconducting character with gaps of 0.81 and 0.62 eV, respectively. The absolute values of the band gap energies are underestimated in comparison with the values obtained from absorbance measurements, but the change with pressure is similar. This a typical feature of DFT calculations, which does not preclude the use of this method for the interpretation of changes induced by pressure in the band structure.^42^ According to our calculations, the effect of the pressure has its reflection not only in a significant reduction of the band gap but also in the change of the semiconducting type of the compound. Whereas for 0.0 GPa, the system cannot be identified as a clear n- or p-type semiconductor, for 3.8 GPa, the density of states profile sets up a scenario where the valence band is almost pinning the Fermi level, revealing a clear p-type semiconducting character of the compound. The reduction of around 0.2 eV in the gap predicted by the theoretical calculations from 0.0 to 3.8 GPa seems to justify the increase in the conductance of several orders of magnitude, as observed in the experiment.

#### Photocatalytic Degradation of Organic Dyes

**Figure 11:**
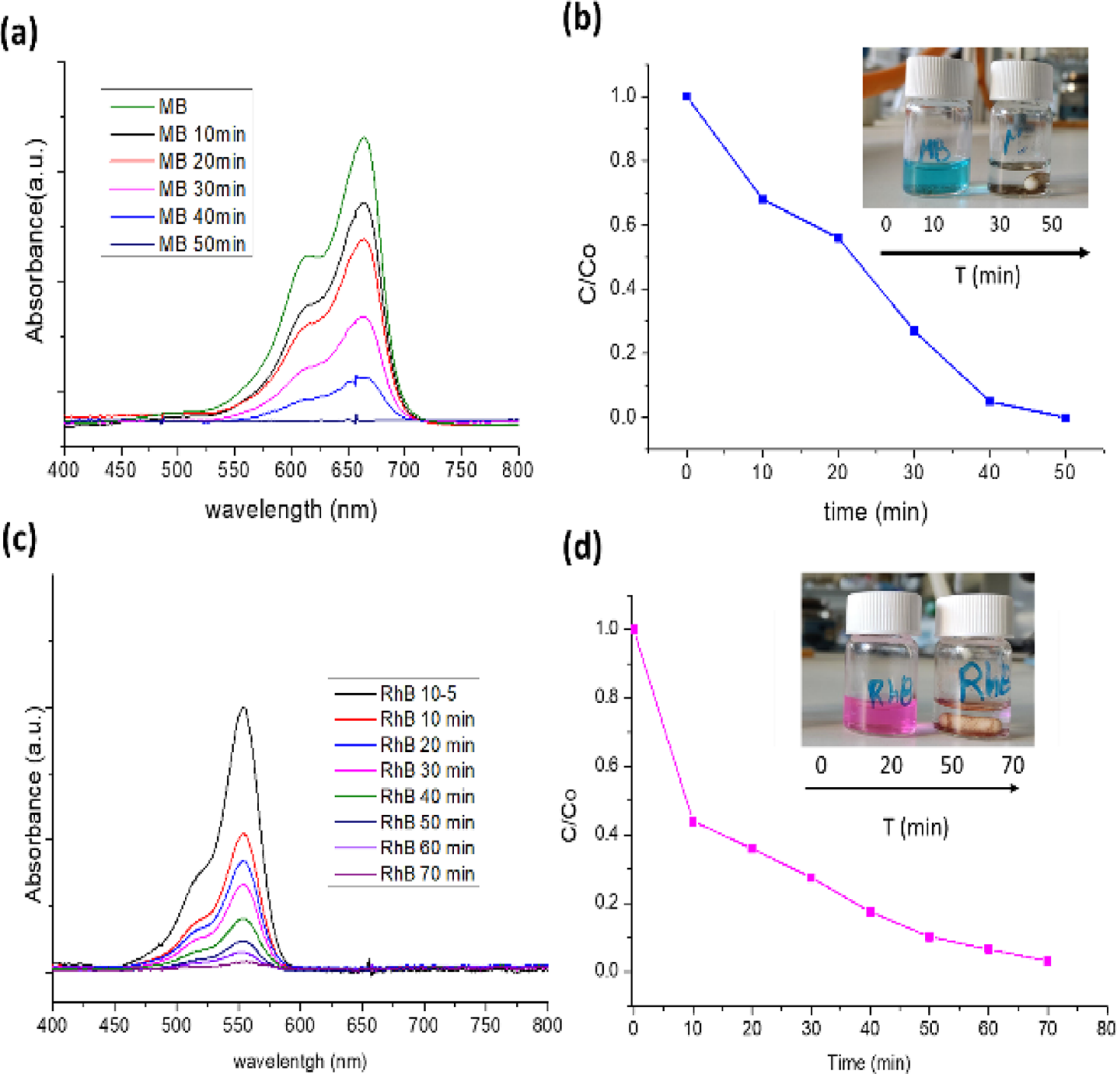
Absorbance vs wavelength for MB (a) and RhB (c) dyes. C/C0 vs time until MB (b) and RhB (d) total degradation.

The effluents from the textile industry are known to contain high levels of possibly toxic organic matter. However, conventional aerobic biological treatment processes are not effective in mineralizing these compounds due to the stability and aromaticity of dyes. Consequently, alternative techniques that are cost-effective and environmentally friendly are being sought.^43,44^ One promising approach is through catalysis, which has been developed for the oxidation of organic pollutants. Taking into account the semiconductor nature of the material under investigation, characterized by an optical band gap of 2.1 eV, and considering the limited research on the photocatalytic properties of Cu(I)-halogen coordination polymers, our objective is to explore its potential for the degradation of persistent dyes, specifically methylene blue (MB) and rhodamine B (RhB), in aqueous solutions.^31,45−48^ To quantify the absorbance of degraded dye over time, we prepared five standard solutions of organic dyes with concentrations ranging from 0 to 10^–5^ M and generated corresponding calibration lines based on their absorbance (Figure S27). For each experiment, a mixture of 2 mg of **CP1** and 2 mL of a 10^–5^ M aqueous solution of the target dye was excited with a wavelength of 300–600 nm. At the start of the reaction (*t* = 0), the UV–visible spectrum showed intense absorption peaks at 660 nm for MB and 550 nm for RhB (Figure 11a,c). Over time, the photocatalytic reaction caused a continuous decrease in absorption peak intensity, indicating the degradation of MB and RhB. We analyzed aliquots of the dye every 10 min using UV–visible spectroscopy, observing complete degradation within 50 and 70 min for MB and RhB, respectively (Figure 11a–d).

The degradation of RhB and MB dyes was found to follow first- and zero-order degradation reactions, respectively, as indicated by the linear correlation between concentration (C/C0) and time (*t*) (Figure S27b–d). The degradation rate constants (*k*’s) were estimated to be 0.045 and 0.02 min^−1^ (Figure S28) for RhB and MB, respectively. After complete degradation, the **CP1** was dried and could be reused for up to three cycles with a gradual increase in degradation time (Figure S29). To confirm the stability and purity of the bulk compound following the photodegradation process, SEM images (Figure S30), IR spectra (Figures S31a), and powder X-ray diffractograms were utilized, which showed no changes in the initial structure even after degradation and agitation inside the photoreactor.^44^

Typically, the degradation process of persistent organic dyes via light (UV) involves the excitation of electrons in the catalyst from the valence band (VB) to the conduction band (CB), leading to the formation of positively charged holes in the valence band. These holes and electrons can then migrate to the catalyst’s surface, where they generate radical species (e.g., hydroxyl radicals and superoxide radicals) responsible for the degradation of the dyes.^57^

## Conclusions

The two-dimensional CPs that we present here based on Cu(I)-I double chains belong to a family of exciting and widely studied CPs with interesting optoelectronic properties capable of responding to different external stimuli (temperature, pressure) by reversibly modifying their optical and electrical properties. This modification takes place because of the high flexibility of their chains that allows these compounds, as happens in this case, to withstand cooling to temperatures close to liquid helium and pressures of up to 11 GPa, contracting or elastically deforming without undergoing a phase transformation. Bond contractions modify the luminescence properties by modifying cuprophilic, metal–halogen, and metal–ligand interactions. In addition, the pressure exerted upon grinding or uniaxially pressing generates defects that decrease the intensity of the emission. However, the electrical response of these types of CPs is less studied, and it is very important to expand the research on their electrical behavior under pressure. In this case, we report a drastic increase in its electrical conductivity when applying pressure, either uniaxially or hydrostatically, in contrast to most of the studies published related to these types of materials, which show that uniaxial pressure generates defects and grain boundaries that decrease their conductivity. The hydrostatic pressure exerted on the crystal decreases its optical band gap, which is in accordance with the increase in its electrical conductivity. The experimental data are also corroborated with DFT calculations.

Another fascinating feature of this CP is its ability to act as a photocatalyst against persistent water-soluble dyes, mainly due to its semiconding behavior, adequate optical band gap, and 2D nature. These results open the door to study these materials in this area where they have been scarcely explored.

Finally, it should be noted that this compound can be obtained in a single step at room temperature, without solvents, in just 2 min of reaction, starting from low-cost commercial reagents, which makes it especially attractive at an industrial level. The presented CP is a multifunctional compound that could have significant applications in areas such as sensors and/or catalysis even in extreme conditions.

## Supporting Information Available

Synthetic procedures, X-ray diffraction studies, structural characterization, thermogravimetric studies, pressure studies, optical properties, diffuse reflectance UV–visible spectroscopy, mechanoluminescence studies, electrical conductivity, DFT calculations, photocatalytic studies (PDF)
