# Supplementary material for: 2D Cu(I)-I Coordination Polymer with Smart Optoelectronic Properties and Photocatalytic Activity as a Versatile Multifunctional Material
Source: Inorg Chem. 2023 Jun 30;62(28):10928–39. doi: 10.1021/acs.inorgchem.3c00616 (PMC10354747; doi:10.1021/acs.inorgchem.3c00616)
Supplement: Supplementary file 1 — ic3c00616_si_001.pdf [file ic3c00616_si_001.pdf]

Supporting Information

2D Cu(I)-I coordination polymer with smart opto-electronic properties and photocatalytic activity as versatile multifunctional material

María Murillo[α], Reinhold Wannemacher[β], Juan Cabanillas-González,[β] Ulises R. Rodríguez-Mendoza[γ], Javier Gonzalez-Platas[μ], Akun Liang[σ], Robin Turnbull[σ], Daniel Errandonea[σ], Ginés Lifante-Pedrola[Δ], Andrea García-Hernán[α], Jose I. Martínez[ν], and Pilar Amo-Ochoa[α,±]*

[α]*Dpto. de Química Inorgánica, Universidad Autónoma de Madrid, 28049 Madrid, Spain*

E-mail: pilar.amo@uam.es

[β]*IMDEA Nanociencia Ciudad Universitaria de Cantoblanco 28049 Madrid, Spain*

[γ]*Dpto. de Física. Instituto Universitario de Nanomateriales y Nanotecnología (IMN). MALTA Consolider Team. Universidad de La Laguna, Avda. Astrofísico Fco. Sánchez s/n, La Laguna, Tenerife, E-38204, Spain*

[μ]*Dpto. de Física. Instituto Universitario de Estudios Avanzados en Física Atómica, Molecular y Fotónica (IUDEA). MALTA Consolider Team Universidad de La Laguna Avda. Astrofísico Fco. Sánchez s/n, La Laguna Tenerife, E-38204, Spain.*

[σ]Dpto de Física Aplicada-ICMUV-MALTA Consolider Team, Universitat de Valencia, c/Dr. Moliner 50, Burjassot (Valencia) 46100, Spain.

[Δ]Dpto. Física Aplicada, Universidad Autónoma de Madrid, 28049 Madrid, Spain.

[ν]Dpto. Surfaces, Coatings and Molecular Astrophysics, Institute of Material Science of Madrid (ICMM-CSIC), University Campus of Cantoblanco, ES-28049 Madrid, Spain

[±]Institute for Advanced Research in Chemical Sciences (IAdChem), Universidad Autónoma de Madrid, 28049 Madrid, Spain

Contents for the supporting information

S1. Experimental section and synthethic procedures
S2. X-ray diffraction studies
S3. Structural Characterization
S4. Thermogravimetric Studies
S5. Pressure studies
S6. Optical properties
S7. Diffuse reflectance UV-visible spectroscopy
S8. Mechanoluminescence Studies
S9. Electrical Conductivity
S10. DFT calculations
S11. Photocalytic studies

S1. Experimental Section and synthetic procedures

ATR-FTIR spectra from 4000 to 650 cm^{-1} were collected using a PerkinElmer 100 spectrophotometer with a universal ATR sampling accessory.

Elemental analyses were conducted by means of a LECO CHNS-932 Elemental Analyzer.

Powder X-ray diffraction data were done using a Diffractometer PANalytical X'Pert PRO with a θ/2θ scanning monochromator and X'Celerator fast detector and 1° primary monochromator for $K_{\alpha 1}$. The samples were performed by scanning θ, from 3-50 degrees, with a time per increment of 100^0 s and an angular increase of 0.0167.

Thermogravimetric analysis was conducted combined with differential thermal analysis (TGA-DTA) using a Pt sample holder on a "TA Instruments Q500 thermobalance oven". The experiments were performed by heating the samples from 25 to 1000 °C at a rate of 10 °C/min, while maintaining a nitrogen gas flow rate of 90 mL/min.

Diffuse reflectance spectra were collected from 100 - 1000 nm with a "UNICAM UV/Vis spectrophotometer". Energy gaps were calculated based on the spectra with Kubelka-Munk function.

To obtain the **Photoluminescence spectra**, we positioned the powdered samples within the recess of an aluminum plate installed on the cold finger of an Oxford Instruments Optistat continuous flow cryostat, which was operated with liquid helium. The spectra were then recorded over a temperature range spanning from room temperature to 9K. The photoluminescence was excited using a "Teem Photonics" 355 nm passively Q-switched Nd:YAG laser, then dispersed by an "Acton SP2500" spectrometer (f=500mm), and detected by either a liquid nitrogen-cooled Princeton Instruments Spec10:400BR CCD camera or a low dark current hybrid photomultiplier (PMA 06, PicoQuant) that were both connected to different exit ports of the spectrometer. We measured PL lifetimes using time-

correlated single-photon counting (TCSPC) with Picoquant TimeHarp TCSPC and multi-channel scaling boar, and fitted decays using Picoquant Fluofit software.

Luminescence at high pressure.

In-situ luminescence high pressure measurements have been recorded exciting at 532 nm with a laser diode and detecting with a Raman spectrometer coupled to a microscope (Renishaw inVia), equipped with an ultra-high sensitivity CCD camera. A 20x SLWD objective allows laser spots of less than 5 µm of diameter. A non-commercial diamond anvil cell (DAC) designed at the University of Paderborn (Germany) was used for high pressure measurements, which allow to attain static pressures up to 40 GPa. For the hydrostatic chamber, two diamond anvils and a 200 µm Inconel pre-indented gasket to 70 µm, with a hole of a diameter of 150 µm was used. To calibrate the pressure measurements, we used the R-lines of Ruby, and as pressure transmitting medium a mixture of 16:3:1 methanol-ethanol-water which provides quasi-hydrostatic pressures of up to 10 GPa. [1]

To examine the composition of the composite material, **Scanning Electron Microscopy (SEM)** images were obtained using an FEI Verios 460 model equipped with an Energy Dispersed X-ray spectroscopy (EDX) system. Sample preparation involved depositing the powdered compound onto a carbon tape using a pipette.

To evaluate the **electrical conductivity**, we conducted direct current (DC) measurements using a two-probe setup at a temperature of 298 K. Both single crystals and pellets of **CP1** and **CP1'** compounds, prepared at different pressures (1.8, 3.7, and 5.5 GPa for 6 minutes), were utilized in the measurements. The conductivity values were determined by applying voltages within the range of -5.0 to 5.0 mV and averaging the recorded results. Contacts were established using Pt wires with a diameter of 25 µm and graphite paste. The samples were examined using a Quantum Design PPMS-9 apparatus,

and they were connected to an external voltage source (Keithley model 2400 source-meter) and an ammeter (Keithley model 6514 electrometer).

To measure **the absorption coefficient**, under high pressure, as a function of the energy**,** we utilized an optical set-up equipped with two confocal Cassegrain objectives and a "USB4000-UV–VIS" spectrometer from Ocean Optics. The absorption spectra at each pressure were obtained by comparing the intensity of the transmittance through the sample to the transmittance through the transparent pressure medium**.**[2] [3] We used a crystal with a surface of 60 μm x 60 μm and a thickness of 10 μm. We applied high pressure using a DAC (480 μm culet anvils). We employed diamonds transparent up to 5.2 eV. The sample was placed in the center of the pressure chamber (200 μm in diameter) made in a stainless-steel gasket pre-compressed to a thickness of 50 μm. The pressure scale and pressure medium, were the same as in luminescence experiments.[1-4]

We conducted **high pressure transport measurements** up to 6 GPa using a DAC furnished with 500 μm culet diamonds. We employed a similar sample to the one used to measure optical-absorption and worked in a four-points configuration. [5] The sample was placed in pressure chamber with similar characteristics to that of optical experiments but with a 250 μm diameter. To ensure electrical insulation between the gasket and electrodes (5 μm Pt filament), we deposited cubic boron nitride epoxy on the surface of the gasket. We used potassium bromide (KBr) as PTM because of its insulating nature and acceptable quasi-hydrostatic characteristics.[4] Pressure was measure by the same method that in the rest of DAC experiments we performed.[6]

Photo-catalytic Studies

We carried out light irradiation using a photoreactor with a 15W purple LED and maintained the temperature at 20°C. To measure the emission of the LED (range λ=300-

600 nm, integration time CR2-AP + 200 ms, and intensity 20.5674 W/m^2), we employed a Stellarnet model Blue-Wave UV-NB50 spectroradiometer.

Photocatalytic Degradation of organic dyes.

To investigate the photocatalytic effectiveness of **CP1** in breaking down methylene blue (MB) and Rhodamine B (RhB), their degradation was analyzed. In order to achieve this, we employed an Agilent 8453 UV-Visible spectrophotometer to generate a calibration curve for the organic dyes and assess their degradation within the samples.

To quantify the dye degradation as a function of time 2 mg of **CP1** were mixed with 2 mL of an aqueous solution of 10^{-5} M of the corresponding dye and the mixture was subjected to irradiation by the LED at the intensity stated above. Every 10 minutes an aliquot was analysed using UV–visible to total degradation of the MB and RhB up to 50 min and 70 min respectively. After complete degradation, the suspensions are centrifuged and the **CP1** was dried. Ensuring the purity and stability of bulk materials holds significant importance for practical applications. In order to evaluate the material's purity for catalytic investigations, we conducted a comparative analysis of SEM images of CP before and after the photo-degradation of MB and RhB. Furthermore, corresponding infrared (IR) spectroscopy and powder X-ray diffractograms were utilized to confirm the absence of any alterations in the initial structure.

Theoretical methods. In order to compute the density of electronic states, atomistic simulations have been performed within a Density Functional Theory (DFT) scheme by using the plane-wave QUANTUM ESPRESSO simulation package.[7] [8] To obtain accurate total energy, we expanded the one-electron wave-functions on a basis of plane-waves, using energy cut-offs of 500 and 600 eV for the kinetic energy and electronic density, respectively. The ultra-soft pseudopotentials were used to model the ion-electron interaction, while the GGA-PBE parametrization was used to account for the exchange-

correlation (XC) effects. [8] [9] To sample the Brillouin zone (BZ), we used optimal (12x4x6) Monkhorst-Pack grids. Structural models and lattice parameters obtained from the experimental PXRD analyses have been used as starting point of the calculations, revealing a net force acting on each atom below 0.05 eV$Å^{-1}$ with a cell strain below 0.1 GPa for the equilibrium configuration with no hydrostatic pressure applied. Spin-polarized calculations do not reveal any difference w.r.t. the unpolarized cases.

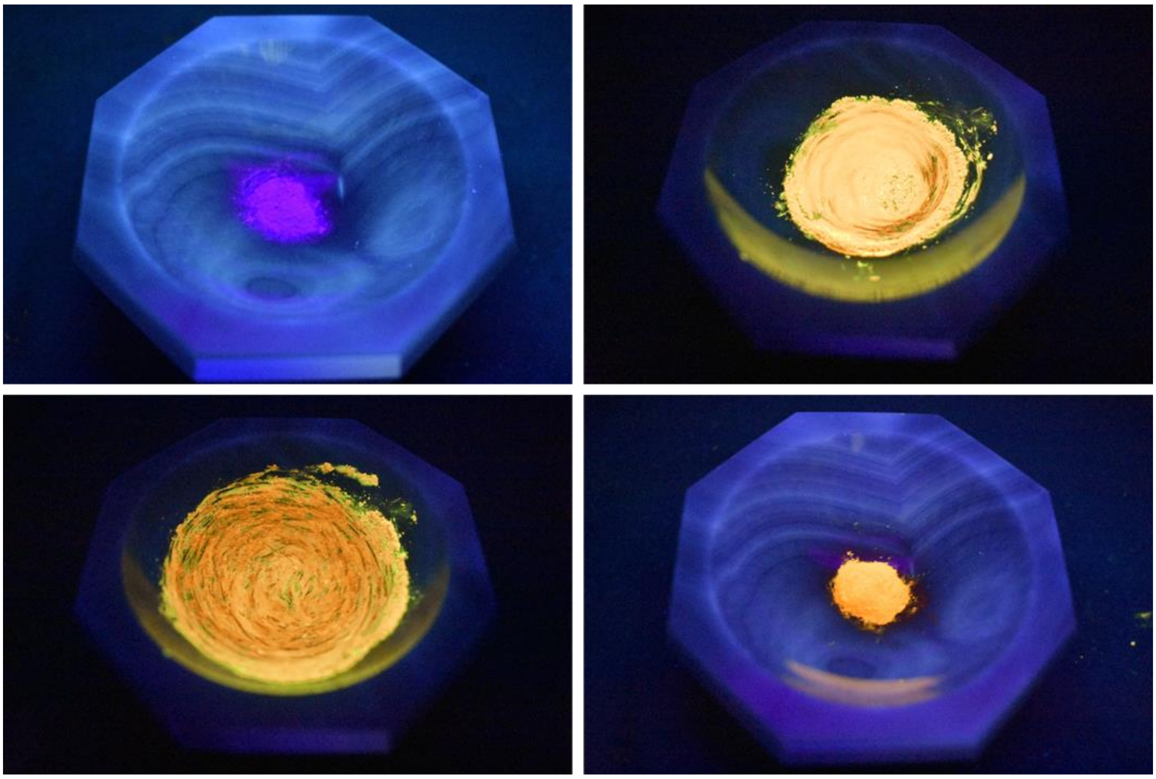

Figure S1. Solvent-free mechanical synthesis of $[Cu_2I_2(PyzF)]n$ (P-1) (**CP1**) polycrystals under UV light (λ_{exc} = 365 nm).

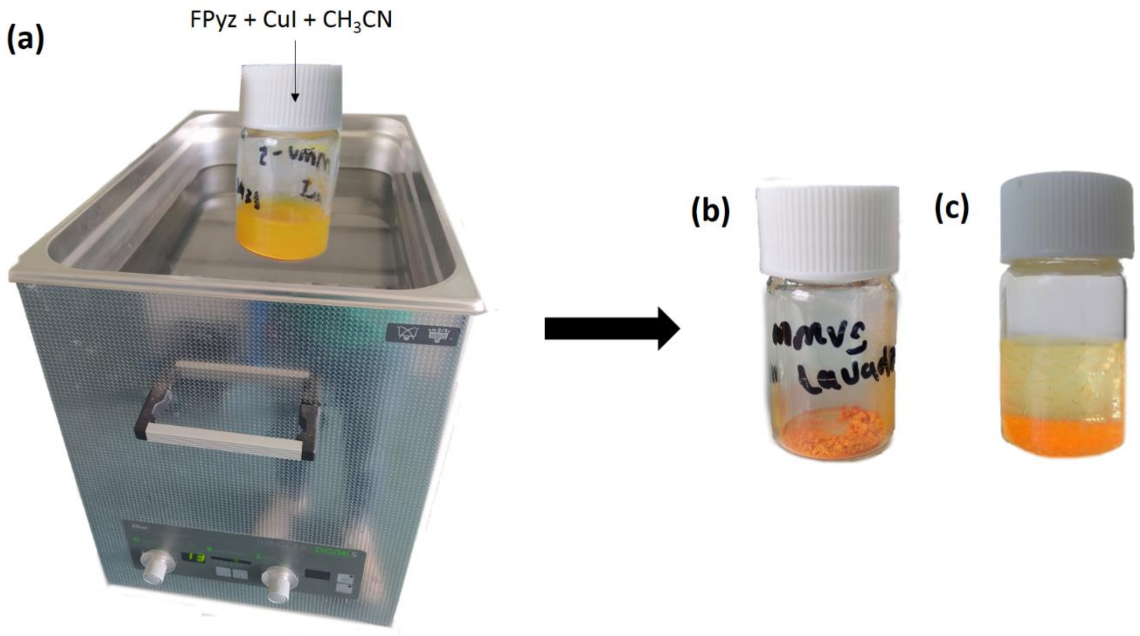

Figure S2. Synthesis of **CP1´** in an ultrasonic bath during 10 minutes at 25ºC (40 KHz) mixing the ligand 2-fluoropirazine (FPyz), copper iodide, and acetonitrile as solvent (**a**). Precipitates an orange powder which is centrifuged and dried (**b**) and in the mother liquid grows single crystals after 24 at 25ºC (**c**).

S.2 X-ray diffraction studies

Table S1. Lattice parameters of compounds **CP1**, and **CP1'.**

**Parameters**	**(CP1)[100K]**	**(CP1´)[100K]**	**(CP1)[200K]**
Formula	$C_4H_3Cu_2FI_2N_2$	$C_4H_3Cu_2FI_2N_2$	$C_4H_3Cu_2FI_2N_2$
$D_{calc.}$/ g cm^{-3}	3.614	3.650	3.580
m/mm^{-1}	11.794	11.912	11.685
Formula Weight	478.96	478.96	478.96
Colour	clear intense orange	clear intense orange	clear intense orange
Shape	prismatic-shaped	prism-shaped	prism-shaped
Size/mm^3	0.08×0.01×0.01	0.11×0.04×0.01	0.11×0.04×0.01
T/K	100.(2)	100.(2)	200
Crystal System	triclinic	monoclinic	triclinic
Space Group	*P*-1	$P2_1$	*P*-1
a/Å	4.221(3)	4.2197(7)	4.2141(4)
b/Å	7.436(5)	12.106(2)	7.5235(7)
c/Å	7.673(6)	8.6893(11)	7.6765(7)
a/°	107.41(4)	90	107.626(3)
b/°	100.35(3)	100.920(6)	100.472 (3)
g/°	99.24(3)	90	99.055(3)
V/Å^3	220.1(3)	435.84(12)	222.16(4)
Z	1	2	1
Measured Refl's.	4641	14026	4620
Indep't Refl's	789	1578	794
GooF	1.111	1.061	1.011
wR_2 (all data)	0.0613	0.0484	0.0917
wR_2	0.0589	0.0467	0.0304
R_1 (all data)	0.0353	0.0267	0.0922
R_1	0.0269	0.0239	0.0319

Table S2. Bond distances and angles of compounds **CP1** and **CP1'**.

Distances	**2D-[Cu_2I_2(Fpyz)]$_n$**		
	**(CP1)$^{200\ K}$**	**(CP1)$^{100\ K}$**	**(CP1')$^{100\ K}$**
Cu-I_{rail}	2.644(2)	2.643(2)	2.626(2)
Cu-I^{i}_{rail}	2.649(2)	2.655(2)	2.652(2)
Δ[Cu-I_{rail}]	0.005	0.008	0.023
Cu-I^{ii}_{rung}	2.622(2)	2.630(2)	2.621(2)
Cu^{i}-I^{i}_{rung}	2.622(2)	2.630(2)	2.623(2)
Cu-N1	2.074(8)	2.094(6)	2.099(9)
Cu-Cu^{i}	2.719(2)	2.707(2)	2.888(2)
Cu-Cu^{ii}	2.971(2)	2.933(2)	2.729(2)
Δ[Cu-Cu]	0.252	0.226	0.159
Cu-I-Cu^{ii}	68.69(5)	67.58(3)	62.36(5)
Cu-I^{ii}-Cu^{ii}	68.69(5)	67.58(3)	62.35(5)
Cu-I^{i}-Cu^{i}	62.09(5)	61.61(3)	66.78(5)
Cu-I^{ii}-Cu^{i}	62.09(5)	61.61(3)	66.72(5)
Cu^{i}-I^{ii}-Cu^{ii}	105.52(6)	105.62(4)	105.99(6)
I^{i}-Cu-I^{ii}	117.91(6)	118.39(4)	113.39(6)
I^{ii}-Cu-I	111.31(6)	112.42(4)	117.74(7)
I-Cu-I^{i}	105.52(6)	105.62(4)	106.26(6)
I-Cu^{ii}-I^{ii}	111.31(6)	112.42(4)	117.55(7)
I^{i}-Cu^{i}-I^{ii}	117.91(6)	118.39(4)	113.39(6)
Dihedral angle	122.11	123.64	124.49

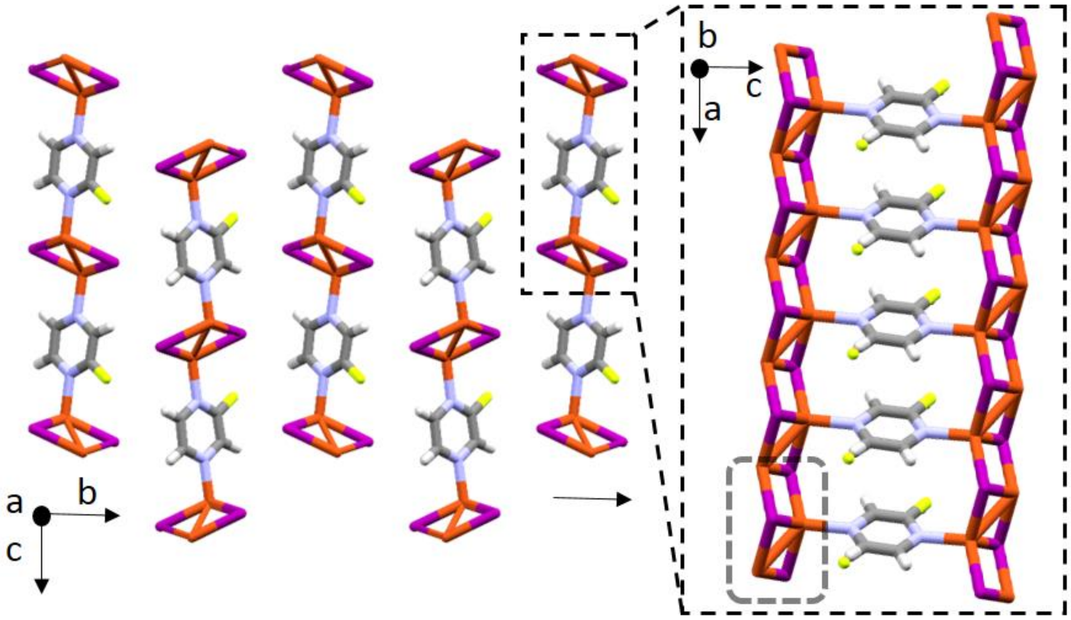

Figure S3. Mercury CCDC representation of 2D [Cu_2I_2(FPyz)]n (**CP1'**) packing. Chain expansion across a-axis.

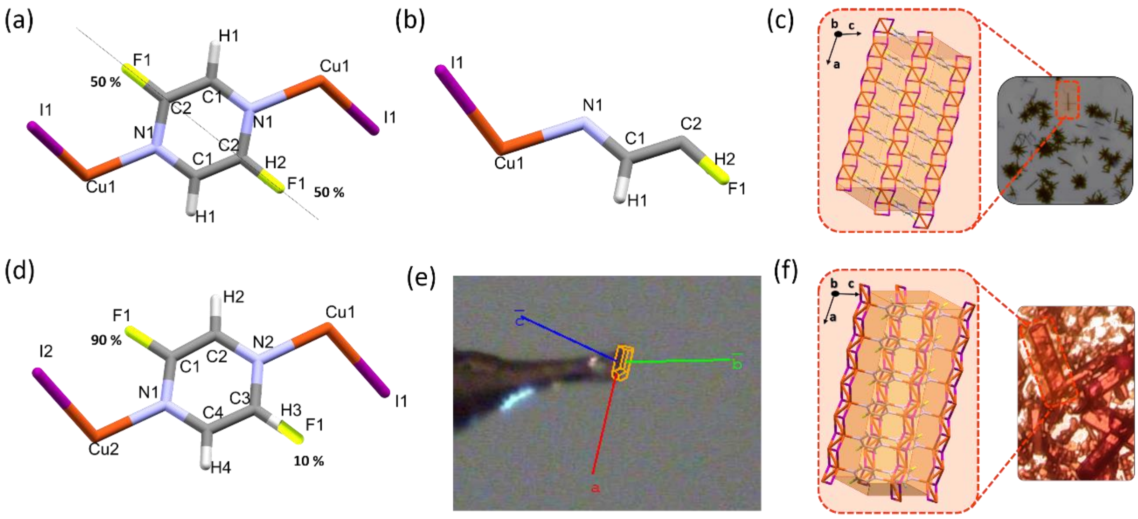

Figure S4. Mercury CCDC representation of **CP1** unit cell with labels (**a**) where two fluorine atoms are represented because the occupancy possibility is 50%, so there is a symmetry plane that makes the asymmetric unit half (**b**). MOPAC Morphology calculation with a microscope image of single crystals of **CP1** (**c**). Mercury CCDC representation of **CP1'** asymmetric unit with labels (**d**) single crystal indexation (**e**) and MOPAC Morphology calculation with a microscope image of single crystals of **CP1'** (**f**).

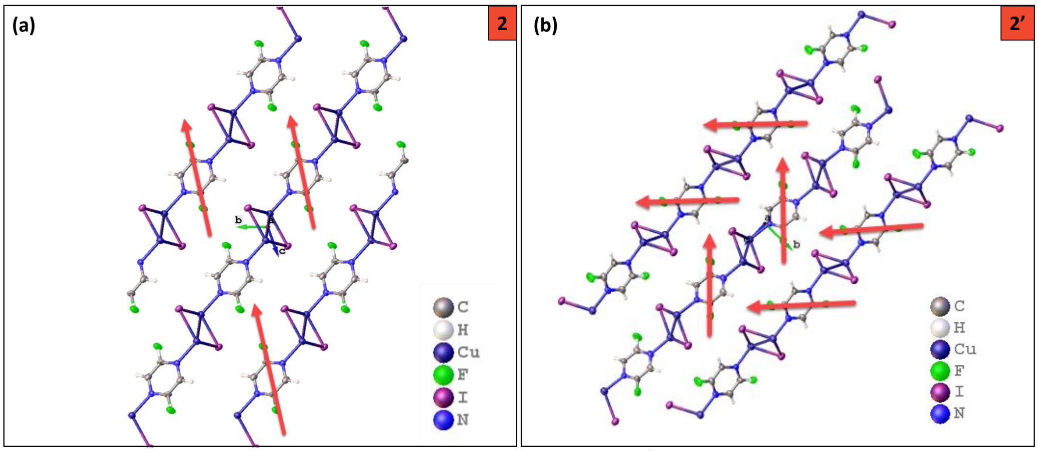

Figure S5. Representation of [Cu_2I_2 (FPyz)]n polymorphs, **CP1**, the one with space group P-1 **(a)** and **CP1’**, polymorph $P2_1$ **(b)** where the different preferred orientation of the fluorine atom can be observed.

S.3 Structural Characterization

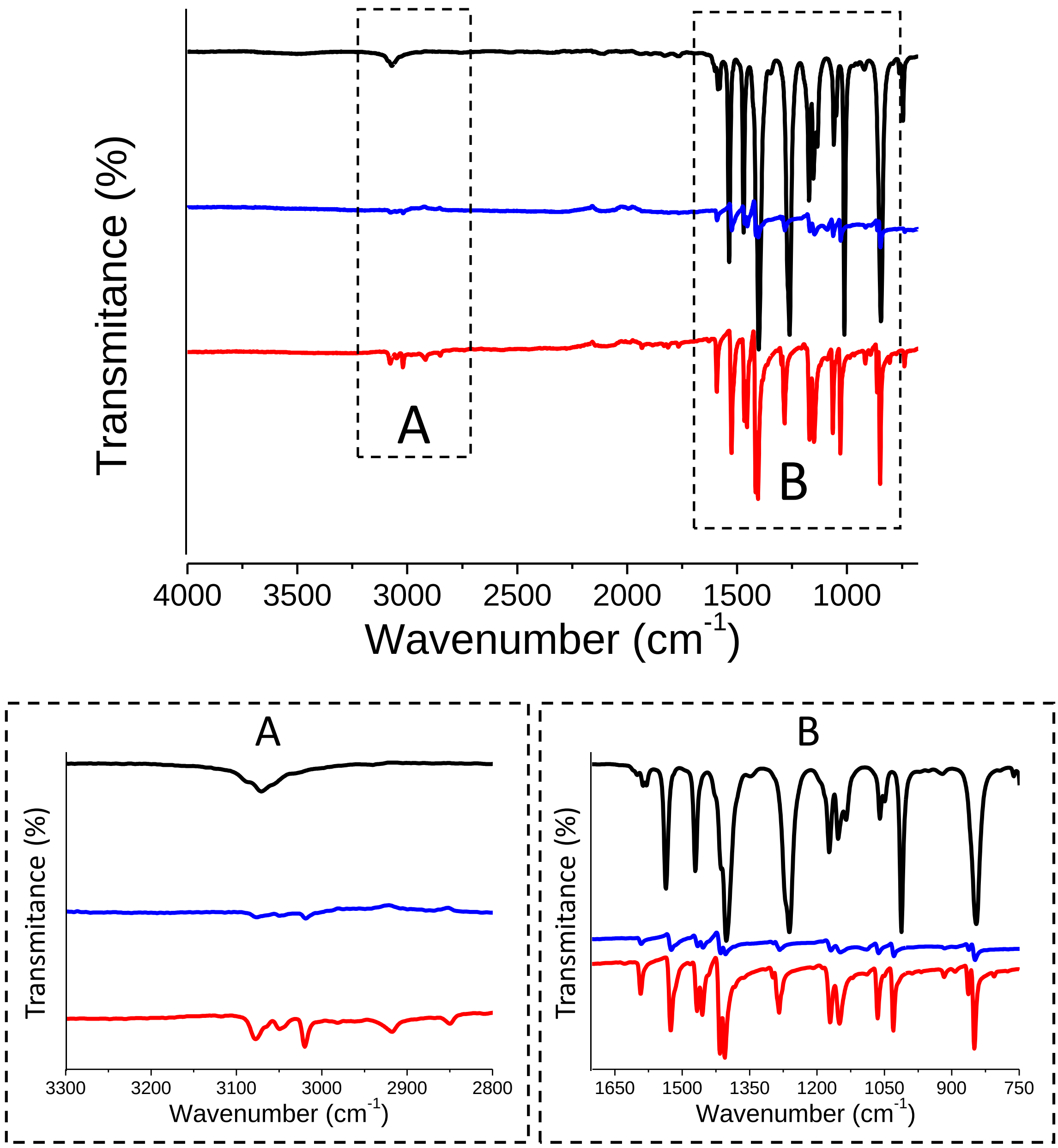

Figure S6. IR Spectrum of Fpyz (black line) and **2D-[Cu_2I_2(Fpyz)]$_n$** (**CP1 = CP1'**) single crystal (blue line) and powder (red line). Boxed figures: Expansion of the wavenumber zone between 3200-2800 cm^{-1} (**A**) and 1750-650 cm^{-1} (**B**).

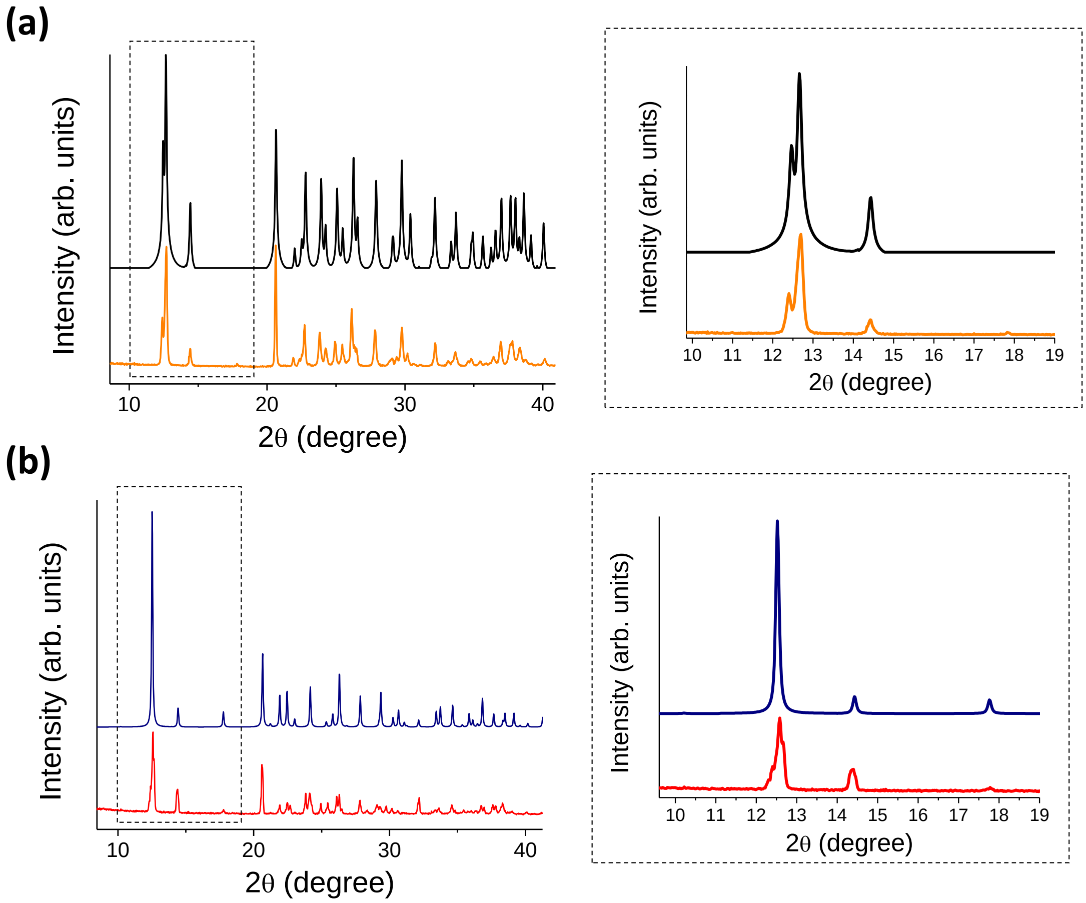

Figure S7. X-ray powder diffractograms (PXRD) of theoretical polymorph P-1 **CP1** (black line) and experimental (orange line) **(a)**. Theoretical polymorph P_{21} **CP1'** (blue line) and experimental (red line) **(b)**. In the right the corresponding zooms.

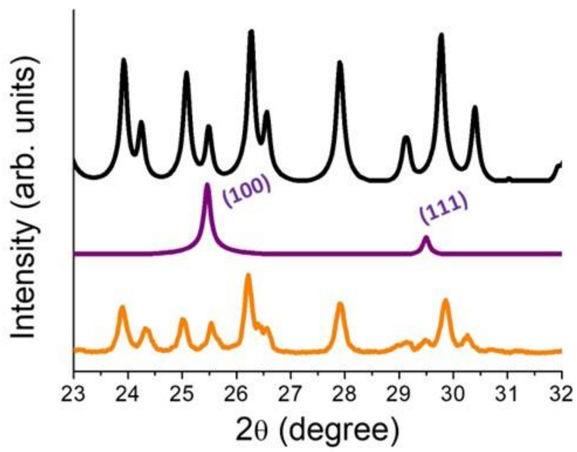

Figure S8. X-ray powder diffractograms (PXRD) zoom of theoretical polymorph P-1 **CP1** (black line) and experimental (orange line) and CuI (violet line).

S4. Thermogravimetric Studies

(a)

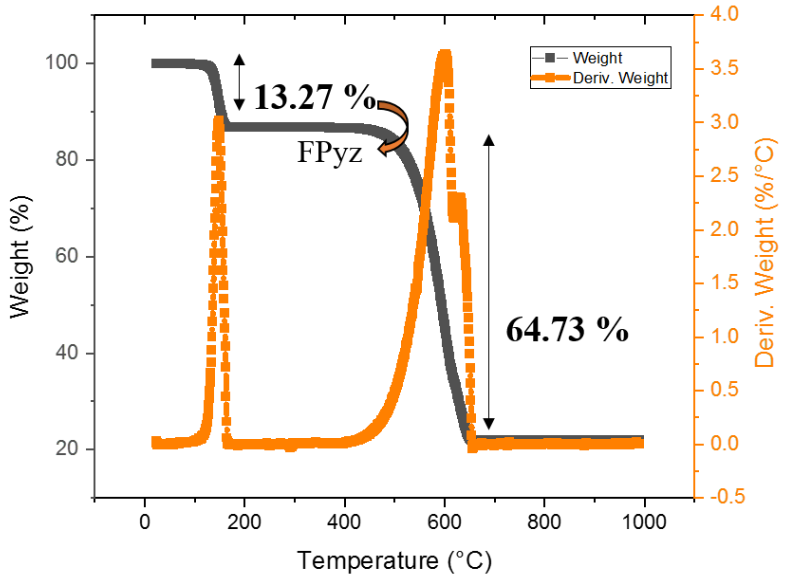

(b)

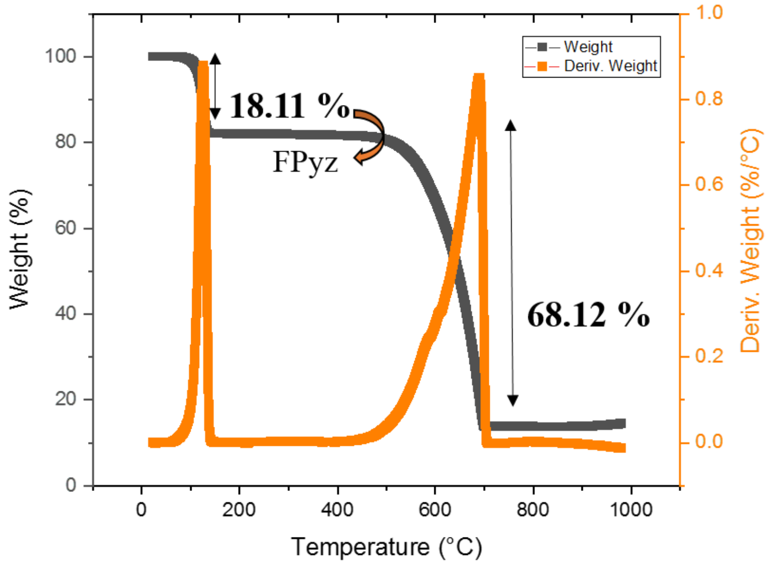

Figure S9. Thermogravimetric analysis of compound **2D-[Cu_2I_2(Fpyz)]$_n$** (**CP1**) (a) and **CP1´**(b), under nitrogen gas with flow rate 90 mL/min and heating rate 10 ºC/min.

S5. Pressure studies

Table S3. Unit cell Parameters vs function of pressure at room temperature of compound **CP1'**.

**Pressure (GPa)**	***a*(Å)**	***b*(Å)**	***c*(Å)**	***β* (°)**	***V*(Å³)**
0.00000(1)	4.2470(3)	12.2087(9)	8.7227(8)	100.713(7)	444.39(7)
0.32(4)	4.2286(5)	11.982(12)	8.6596(16)	100.616(13)	431.3(5)
0.93(4)	4.2048(3)	11.782(7)	8.5714(6)	100.430(6)	417.6(2)
1.55(4)	4.1827(3)	11.605(6)	8.4968(8)	100.277(7)	405.8(2)
2.14(4)	4.1702(3)	11.490(7)	8.4330(8)	100.225(8)	397.7(2)
2.65(4)	4.1553(6)	11.416(13)	8.3731(17)	100.104(15)	391.0(4)
3.17(4)	4.1394(5)	11.316(11)	8.3124(17)	100.015(14)	383.4(4)
3.80(4)	4.1417(4)	11.178(7)	8.2818(11)	100.009(9)	377.6(2)
4.47(4)	4.1339(3)	11.070(6)	8.2350(11)	100.059(9)	371.0(2)

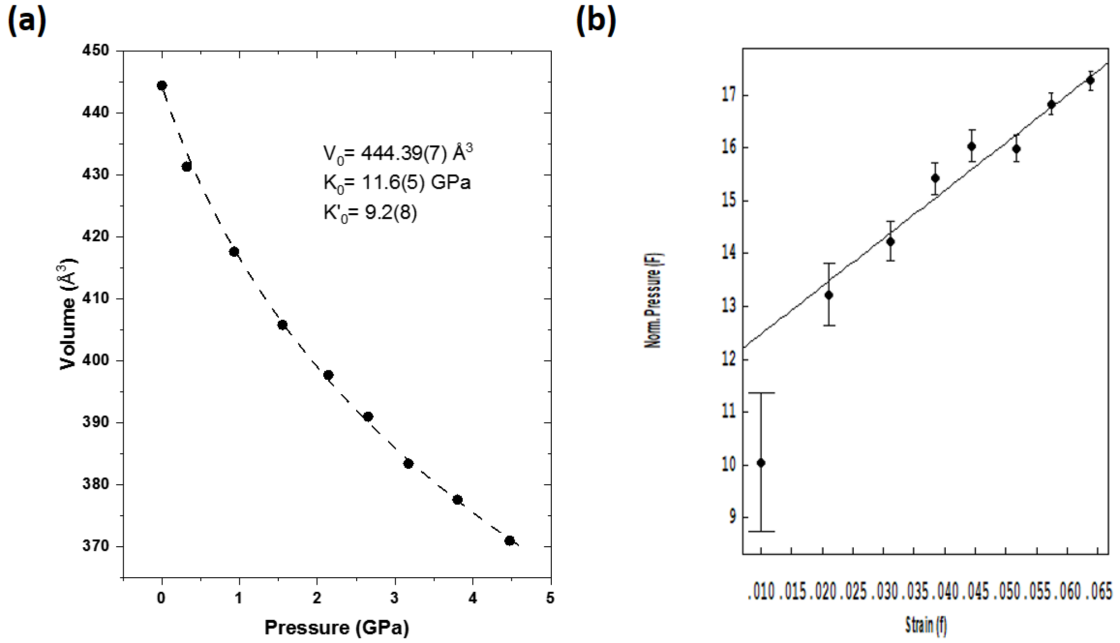

Figure S10. (**a**) Volume vs pressure of **CP1'.** Dash line correspond to the BM3. Error bars are smaller than respective symbols on the plot. (**b**) f-F Plot for volume case.

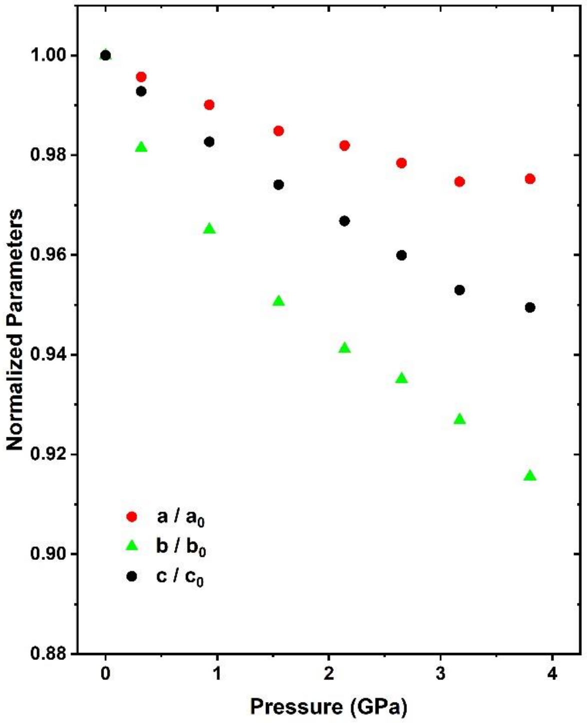

Figure S11. Normalized unit cell parameters vs pressure of **CP1'.** Error bars are smaller than respective symbols on the plot.

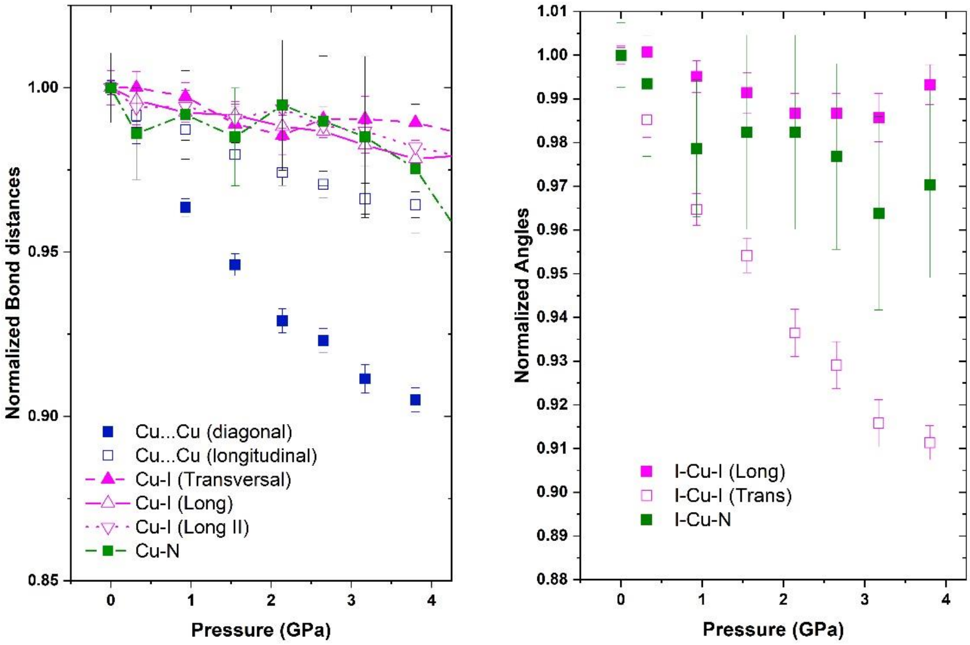

Figure S12. Normalized distances and angles vs pressure of **CP1'.**

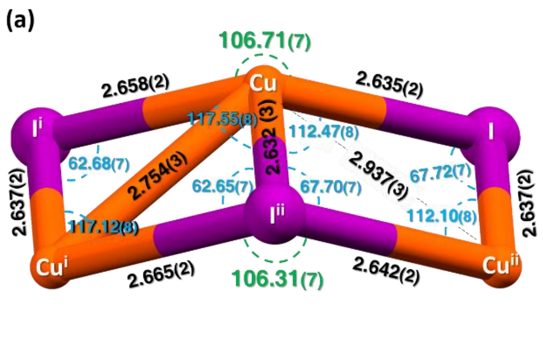

Figure S13. Cu-I chains with atoms labelled, distances (black) and angles (blue) for $[Cu_2I_2(FPyz)]_n$ **CP1'** at 298 K 0 GPa applied (a) and 4.5 GPa (b)

S6. Optical properties

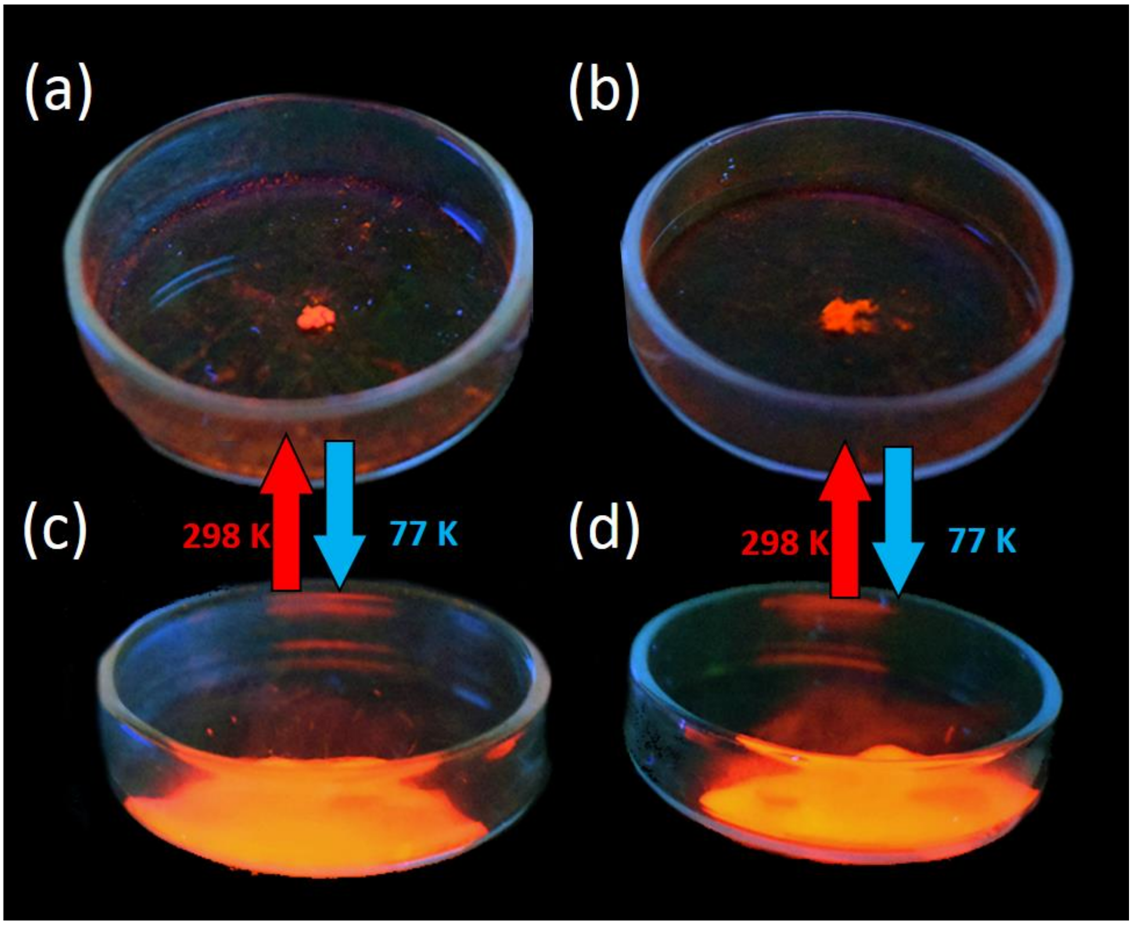

Figure S14. Images under UV light λ = 365 nm of polycrystalline powders at 298 K **CP1** (**a**), **CP1'** (**b**) and at 77 K **CP1** (**c**) and **CP1'** (**d**)

(a)

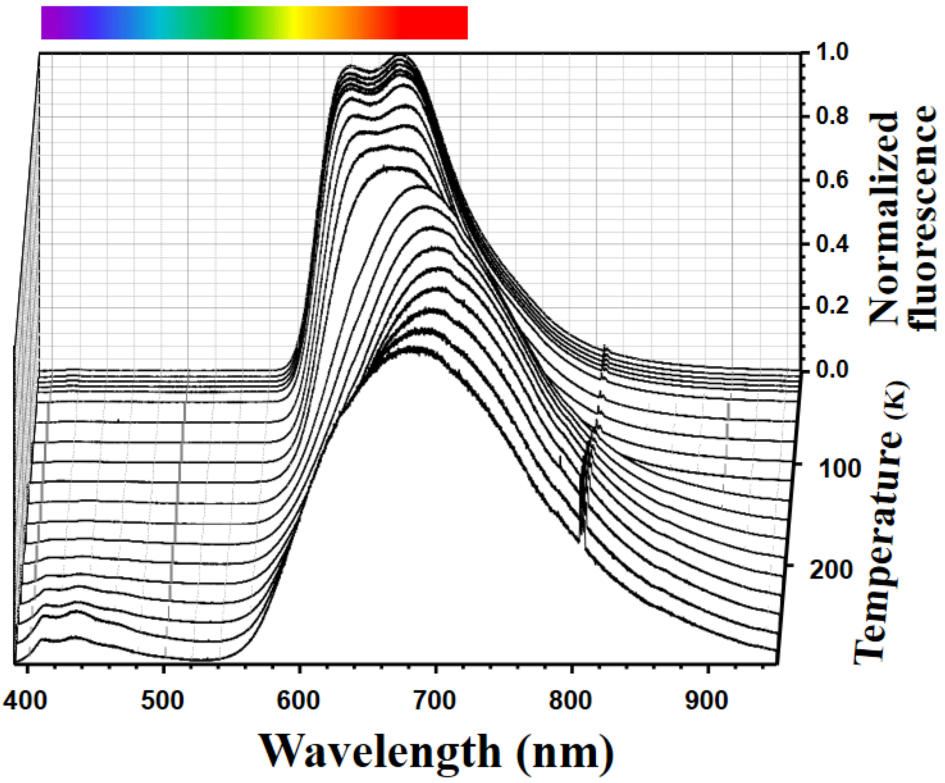

(b)

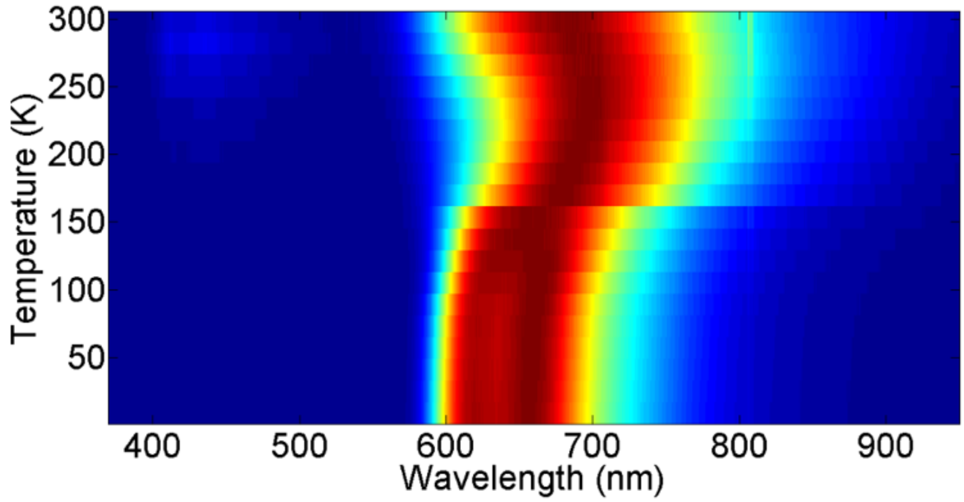

Figure S15. Temperature dependence normalized of photoluminescence emission plots in the solid-state (λ_{exc} = 355 nm) for **CP1** in 3D (**a**) and 2D (**b**).

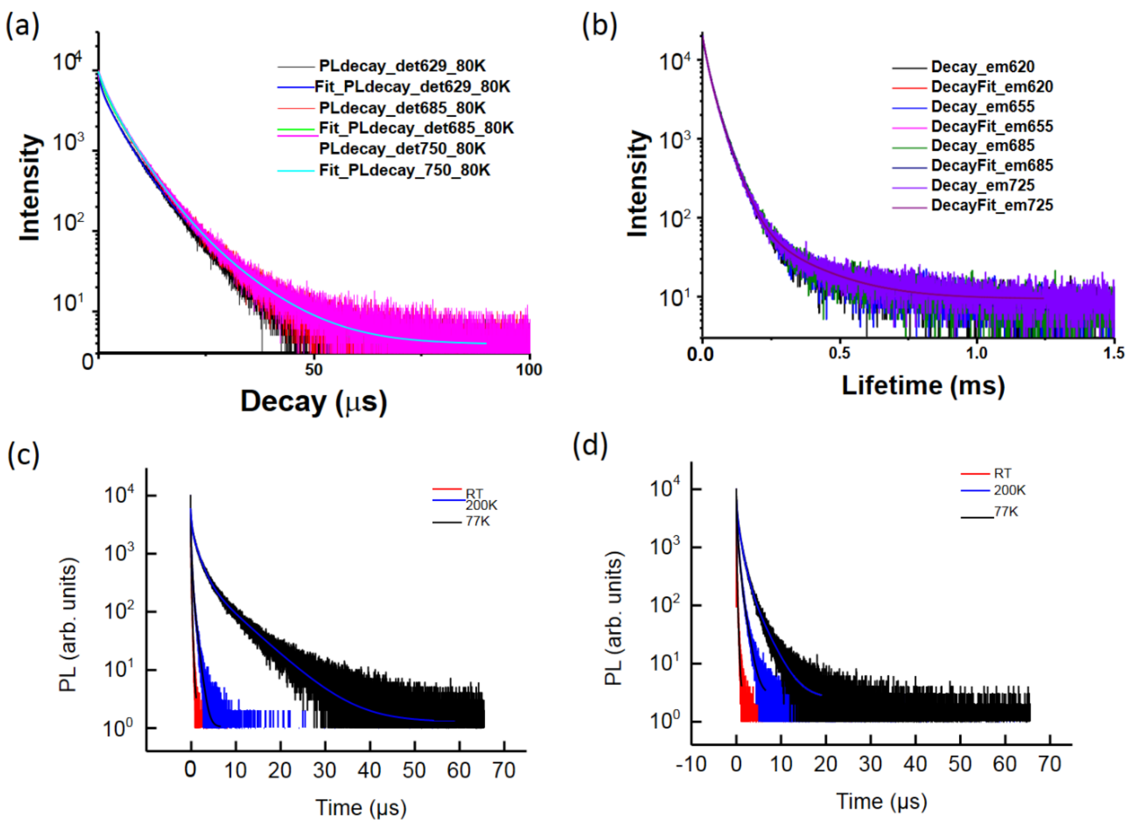

Figure S16. Photoluminescence decay of **CP1** with different wavelengths at 80 K (**a**) and at 9 K (**b**). Photoluminescence decay of **CP1** after 10 minutes of grinding (**c**) and after 10 minutes of hydraulic press (3.8 GPa) (**d**) Temperature dependence of intensity-averaged PL lifetime for **CP1** in the solid-state (λ_{exc} = 365 nm, λ_{emi} = 580 nm).

S7. Diffuse reflectance UV-visible spectroscopy

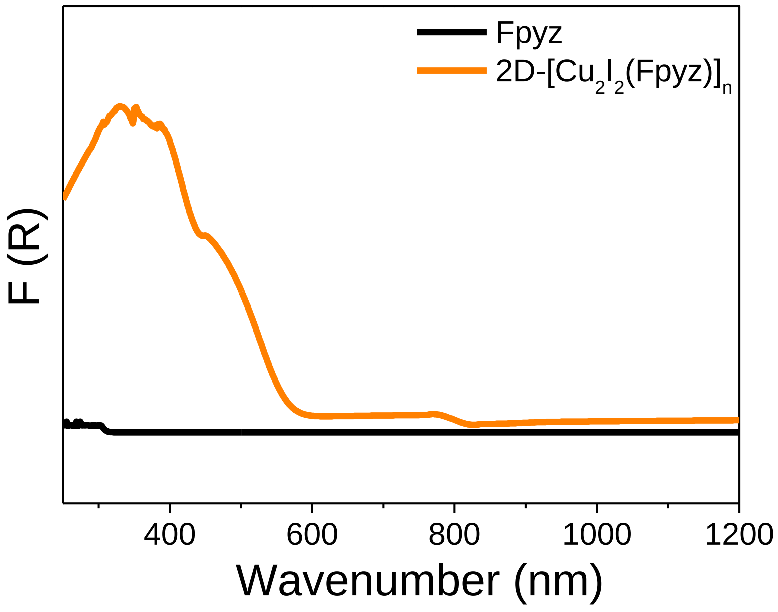

Figure S17. UV-Visible spectrum of **2D-[Cu_2I_2(Fpyz)]$_n$** (**CP1**) (orange line) and FPyz (black line).

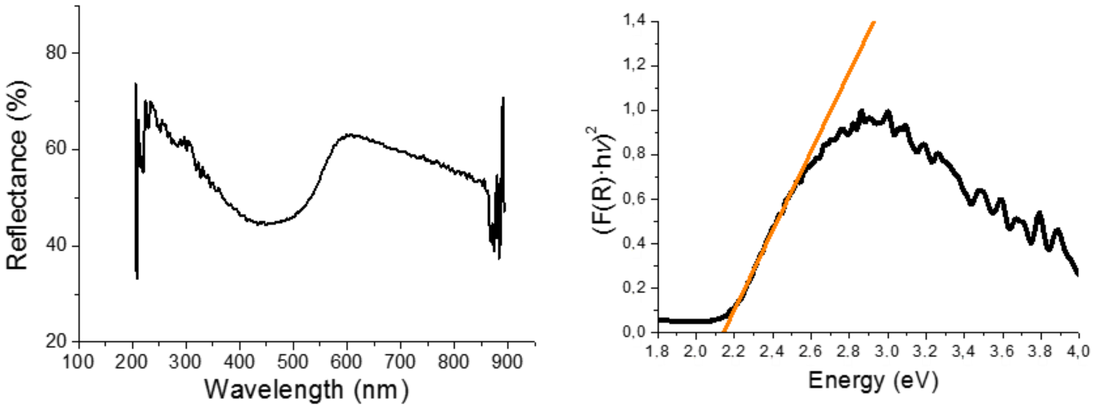

Figure S18. Diffuse reflectance spectra in solid state on the left, and on the right the Kubelka Munk conversion that allows to obtain the gap, for **CP1** [Cu_2I_2(PyrF)]$_n$ a value of Eg = 2.14 eV.

S8. Mechanoluminescence studies

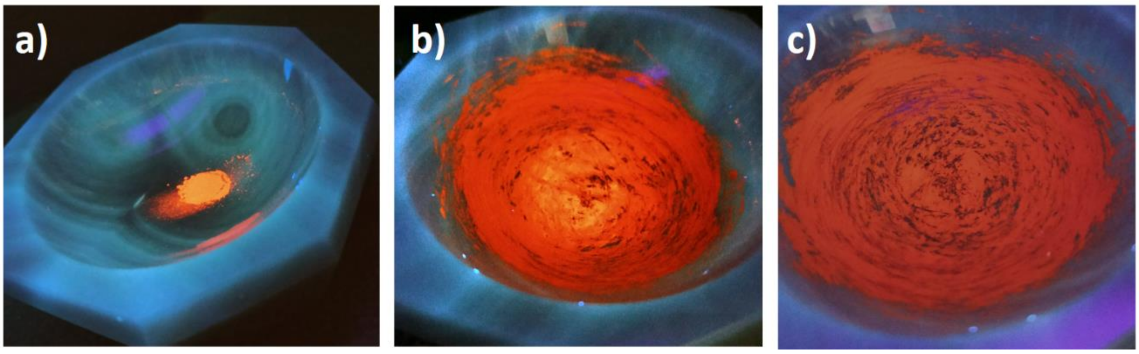

Figure S19. Grinding process of **[Cu_2I_2(FPyz)]n** after mechanical synthesis (**a**), after 2 minutes of grinding (**b**) and after 10 minutes (**c**)

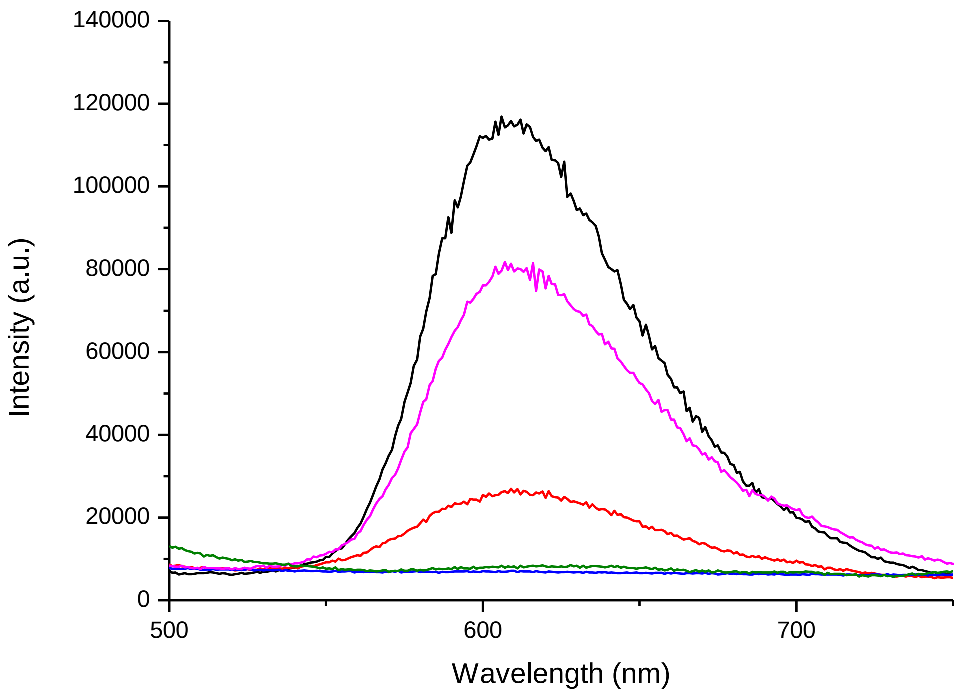

Figure S20. Emission spectra of single crystals **CP1** black line, **CP1** polycrystalline powder after 2 and 10 minutes grinding red and blue lines respectively. **CP1** polycrystaline powder after 5.5 Gpa unialxial pressure 10 minutes**,** green line. With λ_{exc} = 390 nm

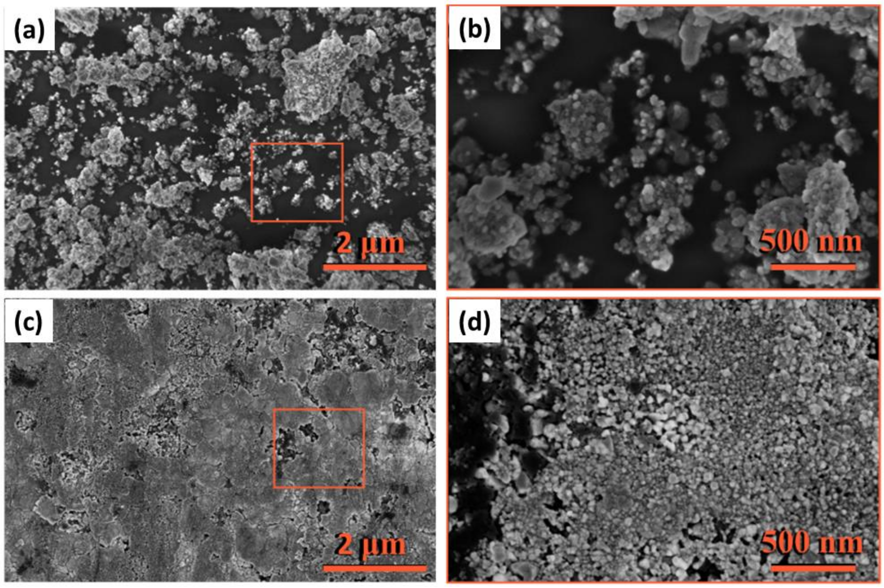

Figure S21. On the left SEM image of **2D-[Cu$_2$I$_2$(Fpyz)]$_n$ CP1** after grinding for 10 minutes (**a**), pressed pellet at 1.8 GPa during 6 minutes (**c**). On the right the respective zoom in orange square (**b,** and **d**).

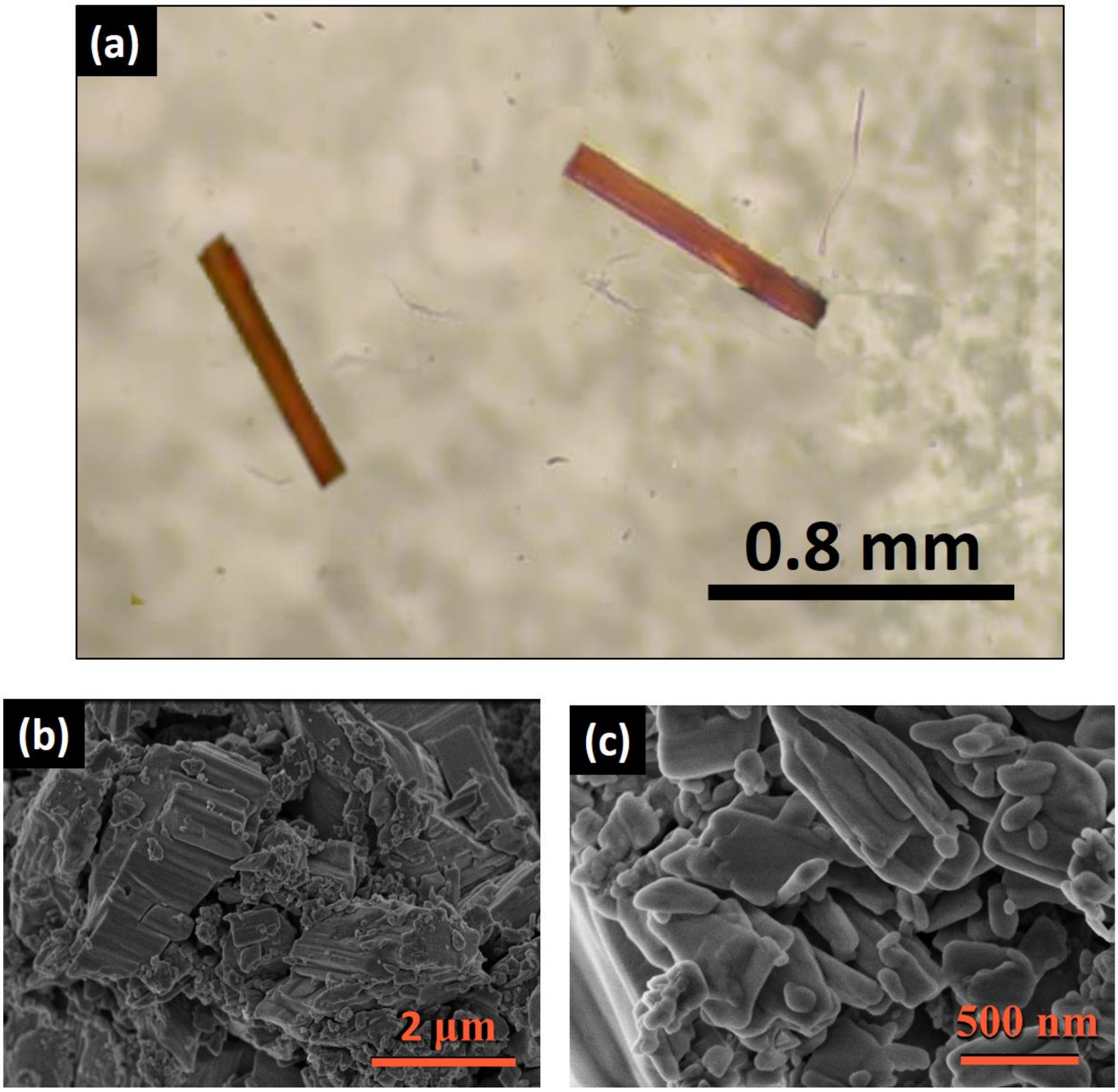

Figure S22. Optical image of **CP1’** single crystal with their dimensions (**a**) and SEM images of **CP1’** with 2 µm scale (**b**) and 500 nm (**c**).

S9. Electrical Conductivity

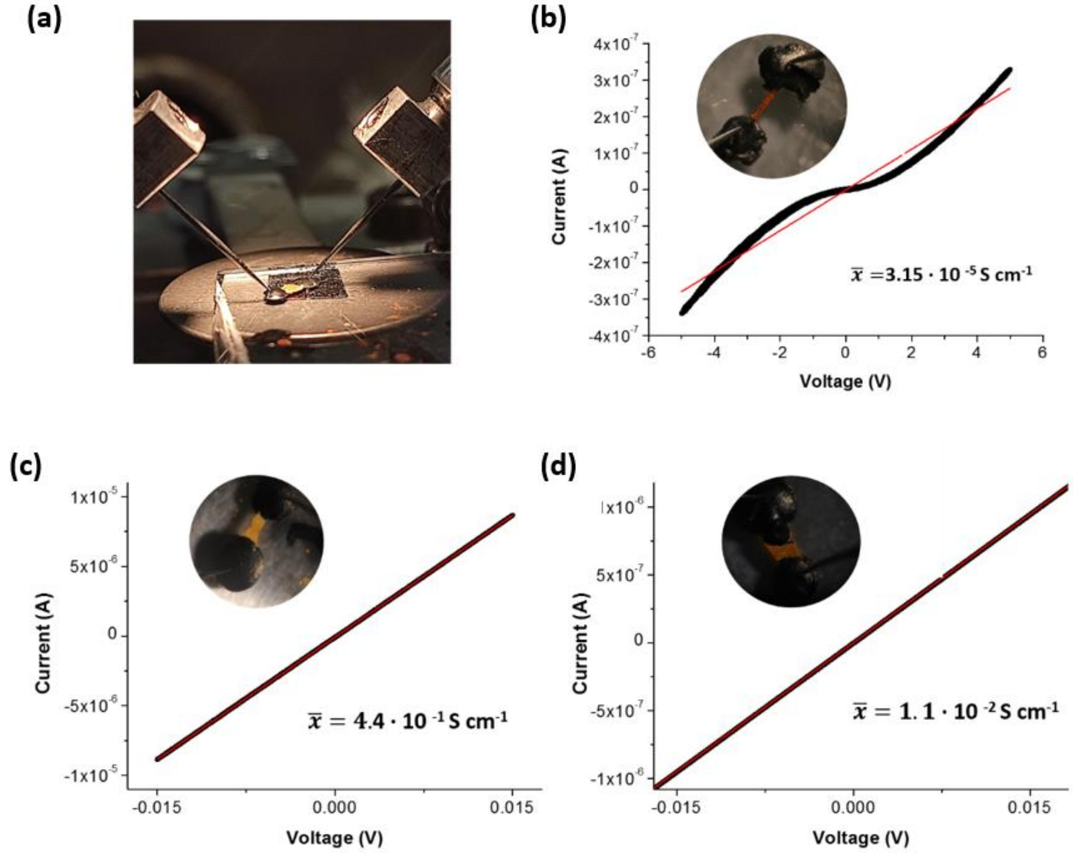

Figure S23. Experimental conductivity assembling (**a**). Graph of current intensity versus voltage of **CP1’** in single crystal (**b**). Pellet pressed at 3.8 GPa of single crystals of **CP1** (**c**) and **CP1’** at 295K (**d**).

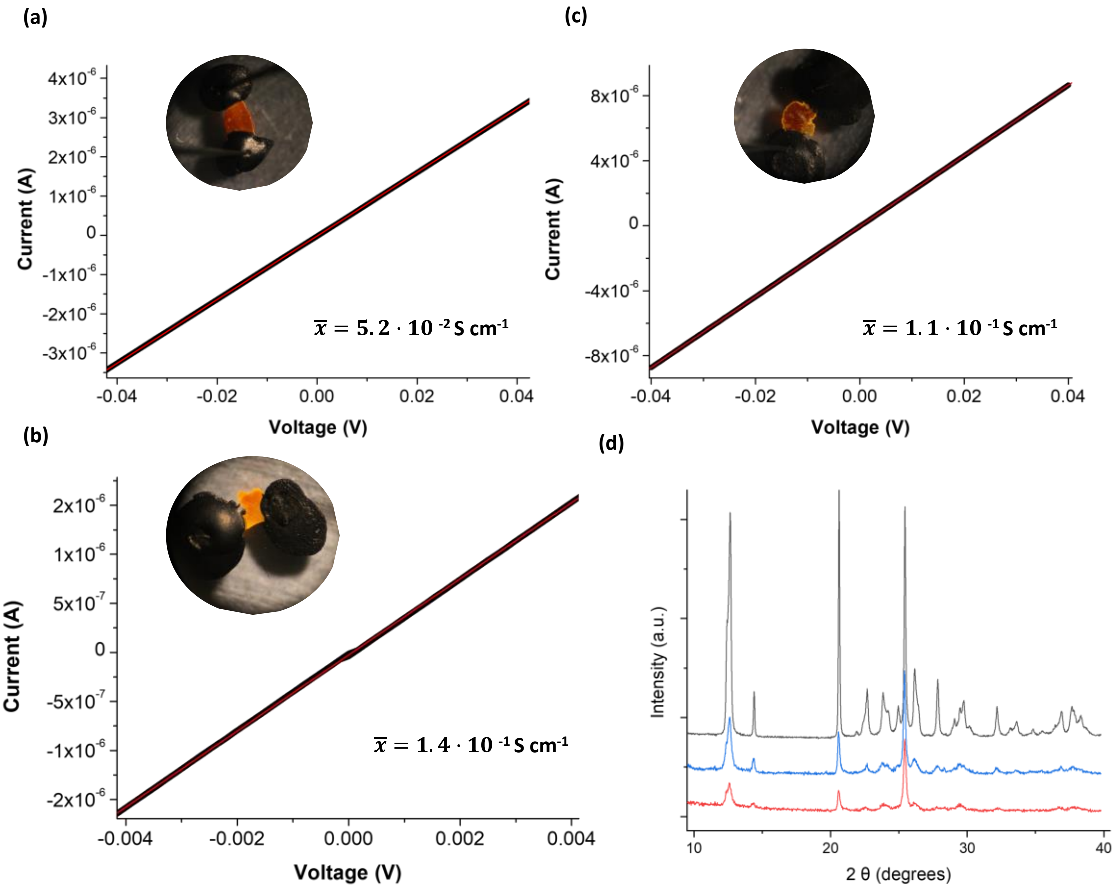

Figure S24. Graph of current intensity versus voltage of **CP1** pressed pellet at 1.8 GPa (**a**), 3.7 GPa (**b**), 5.5 GPa (**c**) and in (**d**) the PXRD at 3.7 GPa (blue line), 5.5 GPa (red line) and the powder of **CP1** after mechanical synthesis (black line)

S10. DFT calculations

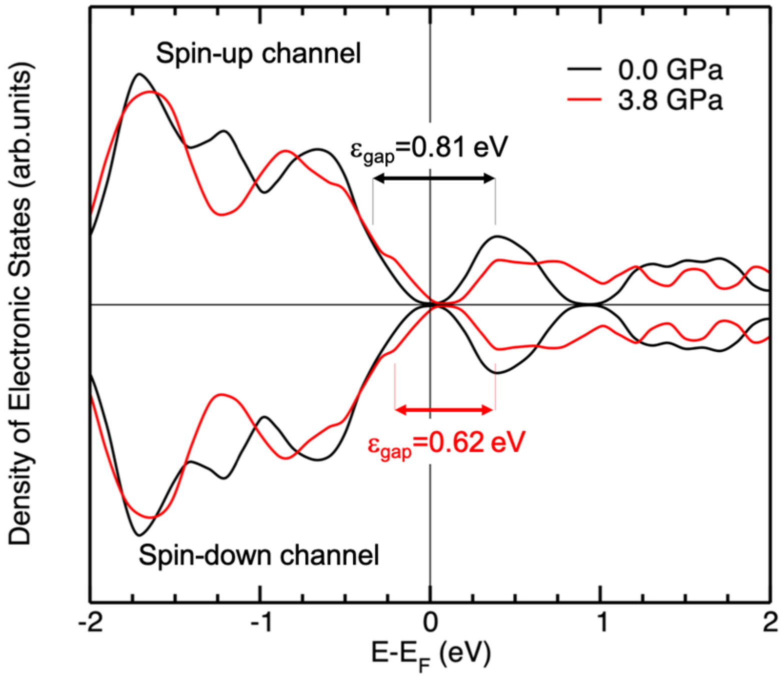

Figure S25. Computed density of electronic states as a function of the energy (referred to the Fermi energy) for the compound at 0.0 (black line) and 3.8 (red line) GPa. Spin-up and spin-down channels are shown separately (top and bottom) with no difference between them. Band-gap values are indicated for each case.

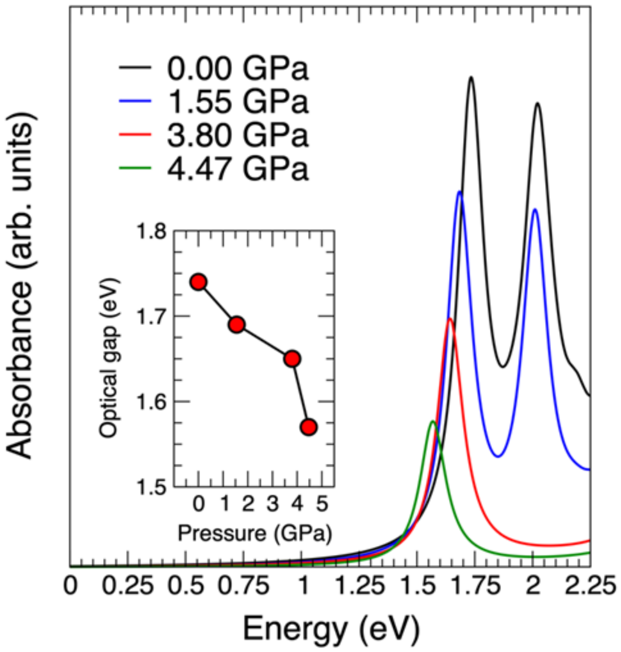

Figure S26. Computed UV-Vis photoexcitation spectra as a function of the photon energy (in eV) for the compound at four different representative external pressures. In the inset we show the optical gap (in eV) obtained from the spectra as a function of the external pressure (in GPa).

In order to shed some light into the optical behavior of the compound for different pressures from the theoretical perspective, and on the basis of the structures experimentally obtained for those pressures, we have used the Time-dependent Density Functional Theory (TDDFT) formalism, as implemented in the QUANTUM ESPRESSO simulation package,[10-11] to compute the excitations and the UV-Vis photoabsorption spectra. Within this formalism, the excitation spectrum is obtained as $I(\omega) \propto Im[\bar{\alpha}(\omega)]$, where $I(\omega)$ is the absorption intensity, and $Im[\bar{\alpha}(\omega)]$ is the imaginary part of $\bar{\alpha}$, the averaged (average of the diagonal elements) dipole polarizability. This dynamical polarizability is represented in terms of the resolvent of its Liouvillian superoperator within TDDFT and evaluated using a non-Hermitean Lanczos method, whose implementation does not require the calculation of virtual states. In the Figure X we show the TDDFT-computed UV-Vis spectra for the compound at four different representative external pressures (0, 1.55, 3.8 and 4.47 GPa). It is interesting to observe how the photoabsorption threshold decreases as the external pressure increases, in excellent agreement with the experimental evidence, yielding TDDFT optical gaps of 1.74, 1.69, 1.64 and 1.57 eV, respectively (see inset within the Figure).

S11. Photocalytic studies

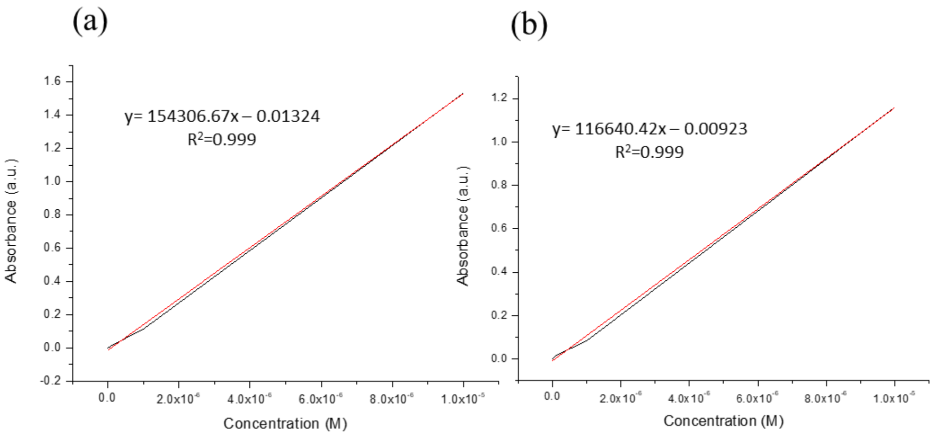

Figure S27. Calibration lines of the organic dyes 0-10^{-5} M. MB (a) and RhB (b)

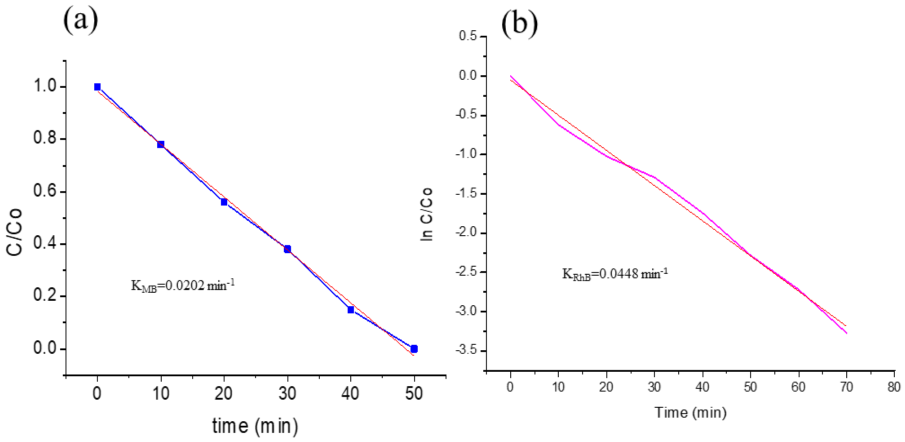

Figure S28. Degradation rate constant (k) for the photocatalysts of MB (a) and RhB (b) with **CP1**

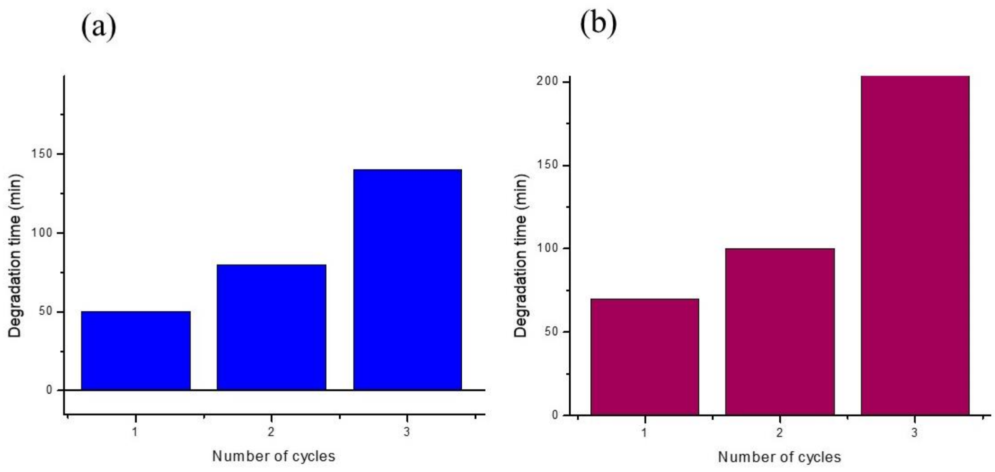

Figure S29: Degradation time versus number of cycles of **CP1** versus MB (a) and RhB (b).

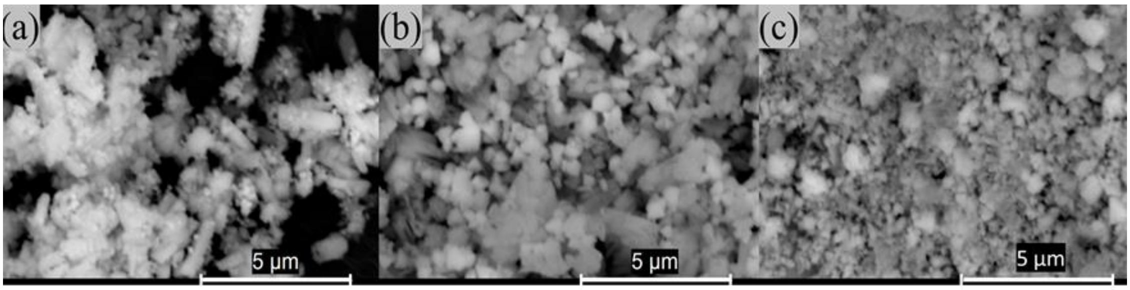

Figure S30. Morphological characterization of the **CP1** (a) before degradation (b) after degradation RhB dye and (c) after degradation MB dye. Scale bars of 5 µm.

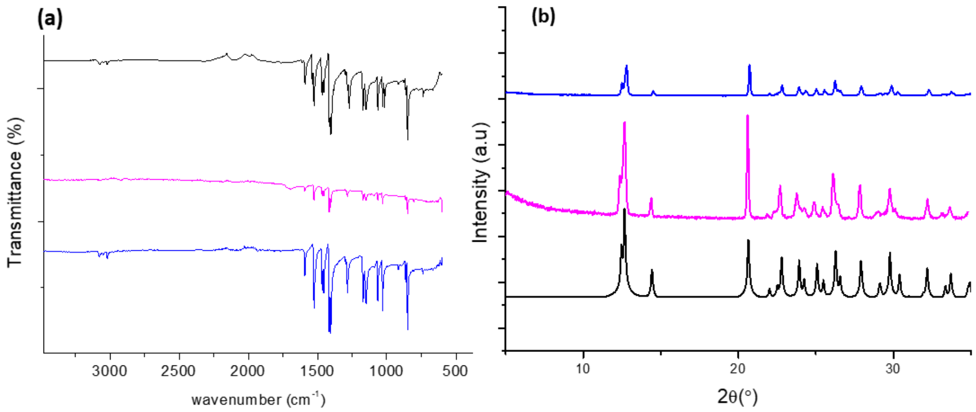

Figure S31. IR Spectrum of Fpyz (black line) and **2D-[Cu_2I_2(Fpyz)]$_n$ (CP1)** powder after degradation with MB (blue line) and powder after degradation with RhB (pink line) (a). DRX-P Spectrum of Cu_2I_2PyrF (black line) and **2D-[Cu_2I_2(Fpyz)]$_n$ (CP1)** powder after degradation with MB (blue line) and powder after degradation with RhB (pink line) (b).

REFERENCES

1. Klotz, S.; Chervin, J. C.; Munsch, P.; Le Marchand, G., Hydrostatic limits of 11 pressure transmitting media. *J. Phys. D: Appl. Phys.* **2009,** *42* (7), 075413.
2. S. X. Li; R. E. Jones; E. E. Haller; K. M. Yu; W. Walukiewicz; J. W. Ager, III; Z. Liliental-Weber; Hai Lu; William J. Schaff, Photoluminescence of energetic particle-irradiated InxGa1−xN alloys. *Appl. Phys. Lett.* **2006,** *88*.
3. Lacomba-Perales, R.; Errandonea, D.; Segura, A.; Ruiz-Fuertes, J.; Rodríguez-Hernández, P.; Radescu, S.; López-Solano, J.; Mujica, A.; Muñoz, A., A combined high-pressure experimental and theoretical study of the electronic band-structure of scheelite-type AWO4 (A = Ca, Sr, Ba, Pb) compounds. *J. Appl. Phys.* **2011,** *110* (4), 043703.
4. Mao, H. K.; Xu, J.; Bell, P. M., Calibration of the ruby pressure gauge to 800 kbar under quasi-hydrostatic conditions. *J. Geophys. Res.: Solid Earth* **1986,** *91* (B5), 4673-4676.
5. Pauw, L. J. v. d., A method of measuring specific resistivity and hall efect of disc of arbitraty shape. *Philips Res. Rep.* **1958,** *13*, 1-9.
6. Yakovenko, A. A.; Chapman, K. W.; Halder, G. J., Pressure-induced structural phase transformation in cobalt(II) dicyanamide. *Acta Crystallogr. Sec. B* **2015,** *71* (3), 252-257.
7. Giannozzi, P.; Baroni, S.; Bonini, N.; Calandra, M.; Car, R.; Cavazzoni, C.; Ceresoli, D.; Chiarotti, G. L.; Cococcioni, M.; Dabo, I.; Dal Corso, A.; de Gironcoli, S.; Fabris, S.; Fratesi, G.; Gebauer, R.; Gerstmann, U.; Gougoussis, C.; Kokalj, A.; Lazzeri, M.; Martin-Samos, L.; Marzari, N.; Mauri, F.; Mazzarello, R.; Paolini, S.; Pasquarello, A.; Paulatto, L.; Sbraccia, C.; Scandolo, S.; Sclauzero, G.; Seitsonen, A. P.; Smogunov, A.; Umari, P.; Wentzcovitch, R. M., QUANTUM ESPRESSO: a modular and open-source software project for quantum simulations of materials. *J. Phys.: Cond. Matt.* **2009,** *21* (39), 395502.
8. Perdew, J. P.; Burke, K.; Ernzerhof, M., Generalized Gradient Approximation Made Simple. *Phys Rev Lett* **1996,** *77* (18), 3865-3868.
9. Vanderbilt, D., Soft self-consistent pseudopotentials in a generalized eigenvalue formalism. *Phys. Rev. B* **1990,** *41* (11), 7892-7895.
10. Malcioglu, B.; Gebauer, R.; Rocca, D.; Baroni, S., TurboTDDFT - A code for the simulation of molecular spectra using the Liouville-Lanczos approach to time-dependent density-functional perturbation theory. *Comp. Phys. Comm.s* **2011,** *182*, 1744-1754.
11. Ge, X.; Binnie, S. J.; Rocca, D.; Gebauer, R.; Baroni, S., turboTDDFT 2.0—Hybrid functionals and new algorithms within time-dependent density-functional perturbation theory. *Comp. Phys. Comm.* **2014,** *185* (7), 2080-2089.
